# Coevolutionary analysis of *Pseudomonas syringae*–phage interactions to help with rational design of phage treatments

**DOI:** 10.1111/1751-7915.14489

**Published:** 2024-06-12

**Authors:** Mojgan Rabiey, Emily R. Grace, Paulina Pawlos, Muscab Bihi, Haleem Ahmed, Georgina E. Hampson, Amna Al Riyami, Leena Alharbi, Rosa Sanchez‐Lucas, Naina Korotania, Maria Laura Ciusa, Olivia Mosley, Michelle T. Hulin, Laura Baxter, Sabrine Dhaouadi, Diana Vinchira‐Villarraga, Robert W. Jackson

**Affiliations:** ^1^ School of Life Sciences, Gibbet Hill Campus University of Warwick Coventry UK; ^2^ School of Biosciences and the Birmingham Institute of Forest Research University of Birmingham Birmingham UK; ^3^ Department of Plant Soil & Microbial Sciences Michigan State University East Lansing Michigan USA; ^4^ Bioinformatics Research Technology Platform University of Warwick Coventry UK

## Abstract

Treating plant bacterial diseases is notoriously difficult because of the lack of available antimicrobials. *Pseudomonas syringae* pathovar *syringae* (*Pss*) is a major pathogen of cherry (*Prunus avium*) causing bacterial canker of the stem, leaf and fruit, impacting productivity and leading to a loss of trees. In an attempt to find a treatment for this disease, naturally occurring bacteriophage (phage) that specifically target *Pss* is being investigated as a biocontrol strategy. However, before using them as a biocontrol treatment, it is important to both understand their efficacy in reducing the bacterial population and determine if the bacterial pathogens can evolve resistance to evade phage infection. To investigate this, killing curve assays of five MR phages targeting *Pss* showed that phage resistance rapidly emerges in vitro, even when using a cocktail of the five phages together. To gain insight to the changes occurring, *Pss* colonies were collected three times during a 66‐h killing curve assay and separately, *Pss* and phage were also coevolved over 10 generations, enabling the measurement of genomic and fitness changes in bacterial populations. *Pss* evolved resistance to phages through modifications in lipopolysaccharide (LPS) synthesis pathways. Bacterial fitness (growth) and virulence were affected in only a few mutants. Deletion of LPS‐associated genes suggested that LPS was the main target receptor for all five MR phages. Later generations of coevolved phages from the coevolution experiment were more potent at reducing the bacterial density and when used with wild‐type phages could reduce the emergence of phage‐resistant mutants. This study shows that understanding the genetic mechanisms of bacterial pathogen resistance to phages is important for helping to design a more effective approach to kill the bacteria while minimizing the opportunity for phage resistance to manifest.

## Graphical abstract

Experimental evolution challenging *Pseudomonas syringae* pv. *syringae* with five phages identified LPS as the most likely target receptor for phage. Mutation of LPS genes was enough to overcome a five‐phage cocktail (5C), but trained phages added to 5C reduced the emergence of phage resistance.

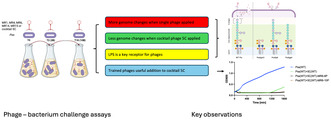

## INTRODUCTION

Bacterial diseases of humans and other animals are typically treated with antibiotic therapy. Historically, plant bacterial diseases were treated with antimicrobials like copper compounds and some antibiotics like streptomycin. However, the rapid emergence and spread of antimicrobial resistance pose a major challenge across all treatments (Sundin & Wang, 2018). Plant disease in particular is problematic due to regulatory limitations that restrict the use of several antimicrobials, leaving many plants susceptible without effective treatment. One potential opportunity is the use of natural and highly specific bacterial viruses, called bacteriophages, as an antimicrobial treatment for pathogens (Brüssow, 2005), offering a promising avenue for addressing this issue.

*Pseudomonas syringae* pathovar *syringae* (*Pss*) causes bacterial canker of *Prunus* species observed as lesions on tree trunks and branches, gummosis and a shot‐hole appearance on leaves, leading to up to 75% tree mortality (Hulin et al., 2018; Spotts et al., 2010). The disease is particularly devastating for commercial growers of cherry (*Prunus avium*). Breeding for resistance or using antibiotics to treat the disease is hampered by the complexity of the disease, caused by different pathovars, and the emergence of resistant bacteria (Hulin et al., 2023; Sundin & Wang, 2018). Chemical control of infected trees is far from ideal given the potential knock‐on effects for surrounding plants and animals in the environment, such as direct toxicity and residue accumulation, and given that many diseased trees are found in close proximity to humans (Cazorla et al., 2002; La Torre et al., 2018).

A potential alternative strategy to treat bacterial plant diseases such as cherry canker is the development of phage biopesticides (phage therapy or phage biocontrol) (Brüssow, 2005; Fischetti et al., 2006; Levin & Bull, 2004). Phage therapy relies on the use of naturally occurring bacterial viruses, called bacteriophages (phages), to counter infections. Lytic bacteriophages kill their bacterial host in order to replicate, and therefore act as an effective antimicrobial agent. Significant research over the past 20 years has successfully explored their use as biological control treatments against bacterial tree pathogens (Grace et al., 2021). The potential of phage against *P. syringae* pathovars *actinidiae* (bacterial canker of kiwifruit), *aesculi* (horse chestnut bleeding canker), *tomato* (bacterial speck disease in tomato) and *porri* (bacterial blight in leek) have been explored as potential biocontrol agents (Di Lallo et al., 2014; James et al., 2020; Rombouts et al., 2016; Skliros et al., 2023; Warring et al., 2022).

Phages initiate infection by recognizing and binding to proteins on the surfaces of bacterial cells. In Gram‐negative bacteria, lipopolysaccharide (LPS) is a common receptor for phages, though other receptors, including outer membrane proteins, pili and flagella, exist (Dowah & Clokie, 2018; Sørensen et al., 2011). LPS is a key component of the outer membrane in gram‐negative bacteria, consisting of lipid A, core and O antigen. It is vital for maintaining a permeability barrier, protecting the bacterium from host defence factors and antibacterial agents, whilst also aiding virulence (Krupa & Srinivasan, 2002). Bacteria can evade phage infection by altering or deleting phage receptors, but in doing so, often compromise other fitness‐related traits such as nutrient uptake, adhesion and virulence (Dy et al., 2014; Mangalea & Duerkop, 2020). As resistance spreads in a population and phage densities decrease, resistance is predicted to be lost over time as a result of relaxed selection (Koskella, 2018). In vitro fluctuation assays show that the rates of spontaneous genetic reversion from resistance to susceptibility can be high (Chaudhry et al., 2018), suggesting that phage sensitivity can (re)emerge within resistant populations. The costs associated with phage resistance vary depending on the mechanism of phage defence employed (Refardt & Kümmerli, 2013). While bacteria can evolve phage resistance in natural environments, this resistance tends to be highly specific due to the high costs associated with resistance mutations in nutrient‐poor natural environments (Buckling et al., 2006). Thus, although resistance may evolve to a particular treatment phage, this is likely to be easily overcome by shifting treatment to an alternative phage genotype or species. Evolving resistance to phage cocktails is more difficult for bacteria due to the constraints of mutational supply and the high costs of carrying multiple resistance mutations (Koskella et al., 2012). In cases where bacteria are challenged with a phage cocktail, the treatment has been shown to amplify the costs of evolved resistance, so bacteria are likely to become sensitive again shortly after treatment is relaxed (Koskella et al., 2012). It is, therefore, important to understand how bacterial resistance emerges and what changes occur under different levels of selection (single phage vs. multiple phage).

In our previous study, 13 MR phages, that lyse and efficiently reduce *Pss* populations, were characterized (Rabiey et al., 2020). Genome sequencing revealed that each phage could be distinguished into one of five genome types; phage MR1 and MR2 had similarities to *Pseudomonas* phage PPPL‐1, which has lytic activity against *P. syringae* pv. *actinidiae*. MR4 had homology to *Escherichia* phage ECBP5, which infects avian pathogenic *Escherichia coli* (APEC). Phages, MR5, MR6, MR7, MR8, MR12, MR16 and MR18, had genome‐wide homology between each other, but less than 50% similarity to *Pseudomonas* phage Φ2, which infects *P. fluorescens* SBW25. MR14 had only 2% sequence similarity to *Yersinia* phage fEV‐1. Phages MR13 and MR15 had similarities to *Pseudomonas* phage ΦPSA1 infecting *P. syringae* pv. *actinidiae* (Rabiey et al., 2020). Single MR phage applications typically led to a quick reduction in bacterial numbers followed by an increase in bacterial population size that then fluctuated, probably due to arms race dynamics (resistant bacteria emerging followed by infective phages emerging). Notably, this was not observed with a mixture, known as phage cocktails, of seven or 13 phages. The bacterial density dropped but very quickly recovered as phage resistance emerged and there was no evidence of arms race dynamics (Rabiey et al., 2020). This might indicate that the phages in the mixture are competing for attachment sites leading to interference between the phages, or alternatively, that the bacterial mutation(s), in altering phage receptor(s) or phage defence systems, was drastic enough to prevent any of the phages attaching to or infecting the cell. To test these hypotheses, the underlying bacterial and phage dynamics and associated evolutionary changes occurring during single‐ and multi‐phage–bacteria interactions were further investigated. Further characterization of how phages interact with their host is, therefore, required to determine how effective they are as robust biocontrol agents. For example, the emergence of bacterial genotypes that are resistant to phages is a major concern, especially if the changes influence bacterial fitness or virulence.

This study aimed to understand how *Pss* and MR phages interact and evolve, if phage resistance emerges and what impacts phage resistance have on bacterial fitness traits and pathogenicity. This helped to identify the major receptor the phages use to attach to *Pss*. Furthermore, experiments to test whether the costs associated with bacterial resistance to phages differ under different conditions were done. The information acquired from these experiments provides the foundation for the rational design of phage cocktails for precise treatment of the *Pss* pathogen.

## EXPERIMENTAL PROCEDURES

### Bacteria and phage culture

*Pseudomonas syringae* pv. *syringae* (*Pss*) strain 9097 grown to an optical density (OD_600_) of 0.2 at 600 nm (≃2 × 10^8^ bacterial cells per mL) was used in this study. Kings Medium B (KB or with added agar [KBA], King et al., 1954) was used to culture *Pss*. Five *Pss‐*infecting phages, collected and characterized by Rabiey et al. (2020), were used in this study: MR1, MR4, MR6, MR14, MR15. Phages were stored and diluted using phosphate‐buffered saline (PBS, Sigma). KB with 0.7% agar was used in the soft top agar overlay for the phage assays.

Phages were amplified by plating 10^6^ plaque‐forming unit (PFU) per mL (to have a clear lawn) stocks with *Pss* on a soft agar overlay plate. After overnight incubation at 27°C, 5‐mL PBS was added onto the plate and incubated at room temperature for 1 h with agitation every 15 min. PBS was removed and filtered through a 0.22‐μm filter to remove any bacteria. Phages were titrated using a spot assay before storage at 4°C.

### Phage killing curve assay

To explore the ability of different phage combinations to lyse *Pss*, killing curve assays were performed using the TECAN SPARK® Multimode Microplate Reader. One hundred microliters of *Pss* and 100 μL of each phage individually or in combination of two, three, four and five, at multiplicity of infection (MOI) of 0.01, were aliquoted to the wells of a Greiner 96‐well flat‐bottomed plate. Positive controls contained 100 μL of *Pss* and 100 μL of KB; negative controls contained 100 μL of PBS and 100 μL of KB. Samples were measured at 600 nm every 20 min for 48 h, preceded by 10 s of shaking. Three replicates were included.

The killing curve assays were then repeated for each of the five phages individually and combination of all five phages (cocktail 5 or 5C) for the duration of 66 h as described above. Bacterial samples were collected at 25, 47 and 66 h to further examine phage resistance in bacterial population when phage was applied individually or in combination. Three colonies per replicate per time point for each treatment were selected and streaked twice on KBA. Frozen stocks were made by incubating each colony in KB for 24 h and storing in an equal volume of 40% glycerol at −80°C.

### Experimental coevolution

Experimental coevolution in vitro was carried out to understand the dynamics of bacterial resistance and phage infection, and to determine whether new infective phage genotypes would emerge. Experimental coevolution of *Pss* with each of the five phages and cocktail of five phages was performed following the method described by James et al. (2020). In brief, 6 mL of KB broth was inoculated with 6 μL of *Pss* and 10 μL 10^6^ PFU mL^−1^ of phage without shaking to hold the bacteria and phage population constant for 10 transfers (Figure S1). Three replicates were performed for each different phage. *Pss*, phage and KB broth were also used throughout the experiment as controls. All the tubes were incubated for 48 h at 27°C after which the tubes were vortexed thoroughly for 10 s and 60 μL of each population (including 60 μL of the control) was removed and transferred to a fresh glass tube containing 6 mL of KB broth. This was continued for 10 transfers and the bacterial population and the phage population were removed and frozen at −80°C in 40% glycerol every second transfer. Bacteria were isolated by spinning down 1 mL of the population, removing the supernatant and washing with 1 mL of PBS twice before freezing them at −80°C. Phages were isolated by filtration of 1 mL of the population through a 0.22‐μm filter. From each replication, phages and bacteria were genome sequenced. A series of bacterial fitness test, including growth curve assay and motility test were carried out. The coevolved phages were also tested against wild‐type *Pss*.

Also, each phage collected at every second coevolution transfer was tested against the bacterial populations to past (two transfers previous), the present transfer (contemporary) and future (two transfers subsequent). Lines of each phage from each second transfer were first streaked onto Petri dishes containing KBA. Then, 20 colonies of each bacterium from every second transfer were streaked across the phage line and the plates were incubated for 48 h before observing for signs of bacterial infectivity. A bacterial colony was classed as sensitive to a phage population if there was any inhibition of growth, otherwise it was classed as resistant. The rate of phage infectivity evolution at each transfer was determined as the proportion of resistant colonies of bacterial populations to past, contemporary and future of sympatric phage populations. This calculation will provide a slope in which both phage infectivity evolution and bacterial resistance evolution can be determined.

### Growth curve assay

To understand phage‐mediated fitness cost of resistance on bacteria, growth curve assays were performed. Overnight cultures of each bacterial generation were centrifuged for 5 min at 2500 *g*, the supernatant removed, and the pellet resuspended in 1 mL of PBS and adjusted to OD_600_ 0.2 and aliquoted onto a Greiner 96‐well flat‐bottomed plate. KB only was used as a negative control. The optical density of each bacterial generation was measured in a TECAN SPARK® Multimode Microplate Reader at 600 nm every 20 min for 48 h, with incubation at 27°C and shaking for 10 s before each reading.

### Spot assay

Spot assays were performed to determine if the ancestral phages could kill any generation of the coevolved bacterial populations. Five microliters of serially diluted phage suspensions (10^2^–10^7^ PFU mL^−1^) was spotted on plates with a soft agar layer containing each coevolved bacterial isolate. The plates were incubated at 27°C for 24 h. After incubation, plates were checked for clearing where the spots of phage were applied.

### Pathogenicity assay of *Pss* isolates on detached cherry leaves

Detached leaf inoculation was performed following Hulin et al. (2023). In brief, freshly picked leaves (1–2 weeks old) were infiltrated with bacterial suspension (2 × 10^6^ CFU mL^−1^) or with 10 mM MgCl_2_ as control, from the abaxial surface using a blunt‐ended 1‐ml syringe. Leaves were then incubated for 7 days. The infiltrated discs were then homogenized in 1 mL of 10 mM MgCl_2_ solution. To assess the bacterial concentration (CFU mL^−1^), a dilution series was prepared and plated to enable the counting of single colonies. Each dilution was tested in triplicate (technical replicates). Each leaf was infiltrated four times using the same strain, and at least three leaves were inoculated with each strain.

### Bacteria DNA extraction

The DNA of each bacterial coevolved generation was extracted using the Invitrogen PureLink Genomic DNA Mini Kit (Carlsbad, USA). DNA concentration and quality were measured via a NanoDrop 2000 (Thermo Fisher Scientific).

### Whole‐genome sequencing

Whole‐genome sequencing was done by MicrobesNG (University of Birmingham, UK) using Illumina‐based services on a NovaSeq 6000 using 2 × 250 bp kits. Forward and reverse sequences were aligned to the *P. syringae pv. syringae* strain 9097 reference genome (accession number: CP026568) or to the MR phages (accession number: MT104465, MT104467, MT104469, MT104474 and MT104475) using BWA‐MEM v0.7.17 (Li, 2013). Alignments were sorted and converted to BAM format using SAMtools v1.10 (Li et al., 2009). Duplicate sequences were removed and read groups were added using the Picard Tools utility v2.21.1 (http://broadinstitute.github.io/picard); MarkDuplicates and AddOrReplaceReadGroups functions respectively. Single nucleotide polymorphism (SNP) calling, indel detection and variant filtration was performed using Genome Analysis Toolkit (GATK) v4.1.5.0 (McKenna et al., 2010). The GATK HaplotypeCaller was used on each isolate with the command –ploidy 1. The GATK VariantFiltration was used to filter SNPs with the following thresholds: QUAL <30.0 (overall quality filter); QD <2.0; SOR >3.0; FS >60.0; MQ <40.0; DP <5.0. Overlapping genomic features between the isolates and reference genome were detected using bedtools intersect command (v2.29.2) (Quinlan & Hall, 2010). Final SNPs were set at a minimum coverage of 30 and a minimum of 90% variant frequency. All SNPs identified in this work had >95% variant frequency.

### Construction of *Pss* markerless mutants

Six genes that were involved in *Pss* coevolution or interaction with phages MR4, MR6 and MR14 were deleted. The genes were as follows: glycosyltransferase family 1 (*gst1*, BKC06_002880, 957 bp), lipopolysaccharide kinase (*lpk*, BKC06_002845, 780 bp), glucose‐1‐phosphate thymidylyltransferase (*gpt*, BKC06_005130, 882 bp), phosphomannomutase/phosphoglucomutase (*pmm*, BKC06_001185, 1398 bp), autotransporter outer membrane beta‐barrel domain‐containing protein (*aom*, BKC06_013135, 2745 bp), ATP‐grasp domain‐containing protein (*agd*, BKC06_020630, 4665 bp). Primers were designed (Table S1) to enable amplification of DNA on either side of the target genes from *Pss* genome sequence (Genbank accessions: CP026568.1), using Geneious (Kearse et al., 2012). Splicing by Overlap Extension (SOE) PCR was done using F1 and R2 primer pair. Each PCR was done in 50‐μL volume using Q5® High‐Fidelity 2X Master Mix (NEB, UK). The PCR conditions were as follows: 98°C for 30 s, 30 cycles of 98°C for 10 s, 72°C for 30 s, 72°C for 30 s and final extension of 72°C for 2 min and hold on 4°C. PCR purification or gel extraction was done to extract DNA, using a PCR Purification Kit (QIAquick®, UK) or Gel Extraction Kit (NEB, UK).

To create the pK18mobsacB deletion construct restriction digest was performed to cut the pK18*mobsacB* plasmid (accession FJ437239) and the SOE DNA. The plasmid was extracted using a Plasmid Miniprep Kit (ThermoFisher Scientific, UK). PCR was conducted in 25‐μL volume containing 9‐μL DNA (either SOE DNA or pK18*mobsacB* plasmid DNA), 2.5 μL of enzyme buffer r3.1 (NEB, UK), 1 μL of each restriction enzyme and 11.5 μL of nuclease‐free water. For the control, nuclease‐free water instead of DNA was used. The PCR reaction was performed at 37°C for 1 h and then PCR products were electrophoresed on 1% agarose gel and PCR purification was done as above.

NEB 5‐alpha *E. coli* competent cells were used for the transformation process with the pK18*mobsacB*‐SOE vector containing SOE DNA insert, following the manufacturer's protocol. pUC19 DNA was used as a control. Lysogeny broth agar (LBA) plates were prepared with kanamycin (50 μg mL^−1^) and X‐Gal (40 μg mL^−1^) and spread with transformation product. The plates were incubated at 37°C for 24 h. The white colonies were plasmid extracted following the plasmid miniprep kit (Invitrogen, UK) instruction. To confirm the insert of the vector in *E. coli* cell, PCR was performed using MR13 primer pair (Table S1).

For conjugation, 1 mL of the *E. coli* containing pK18*mobsacB*‐SOE vector, 500 μL of the bacteria harbouring the pRK2073 helper plasmid and 500 μL of *Pss* 9097 were mixed and centrifuged at 18,000 *g* for 2 min. The pellet was resuspended and spread onto KBA plates. The plates were incubated at 30°C for 24 h. Then, a loopful of the lawn was spread onto KBA plates containing kanamycin and nitrofurantoin (100 mg mL^−1^) and incubated at 27°C for 3–7 days.

A single colony of *Pss* was taken and mixed in 500‐μL sterilized deionized water. A 50‐μL aliquot was spread onto KBA plates containing 10% (w/v) sucrose and incubated at 27°C for 48 h. A single colony was taken from the sucrose plate and spread onto the KBA plate, and then mirrored onto the KBA with kanamycin plate and incubated at 27°C for 2–7 days. To identify putative mutants, the colonies which grew on KBA were PCR tested using F1 and R2 primer pairs and using a gene that was present within the gene of interest. ON culture of confirmed mutants was frozen in 40% glycerol at −80°C.

### Statistical analysis

To evaluate differences in the killing curves and growth curves of the bacterial isolates in the absence and presence of phages, individually or in cocktail 5C, ANOVA test was used at specific time points, that is, at 500, 1000, 1500 and 2000 min. Post hoc Tukey's test was applied to evaluate differences among treatments (*p* < 0.05). All the statistical analyses were carried out in GraphPad Prism 9 (Boston, MA, USA, www.graphpad.com), shown in Table S2.

## RESULTS

### Bacterial resistance to phage occurs in single and multi‐phage treatments

**FIGURE 1:**
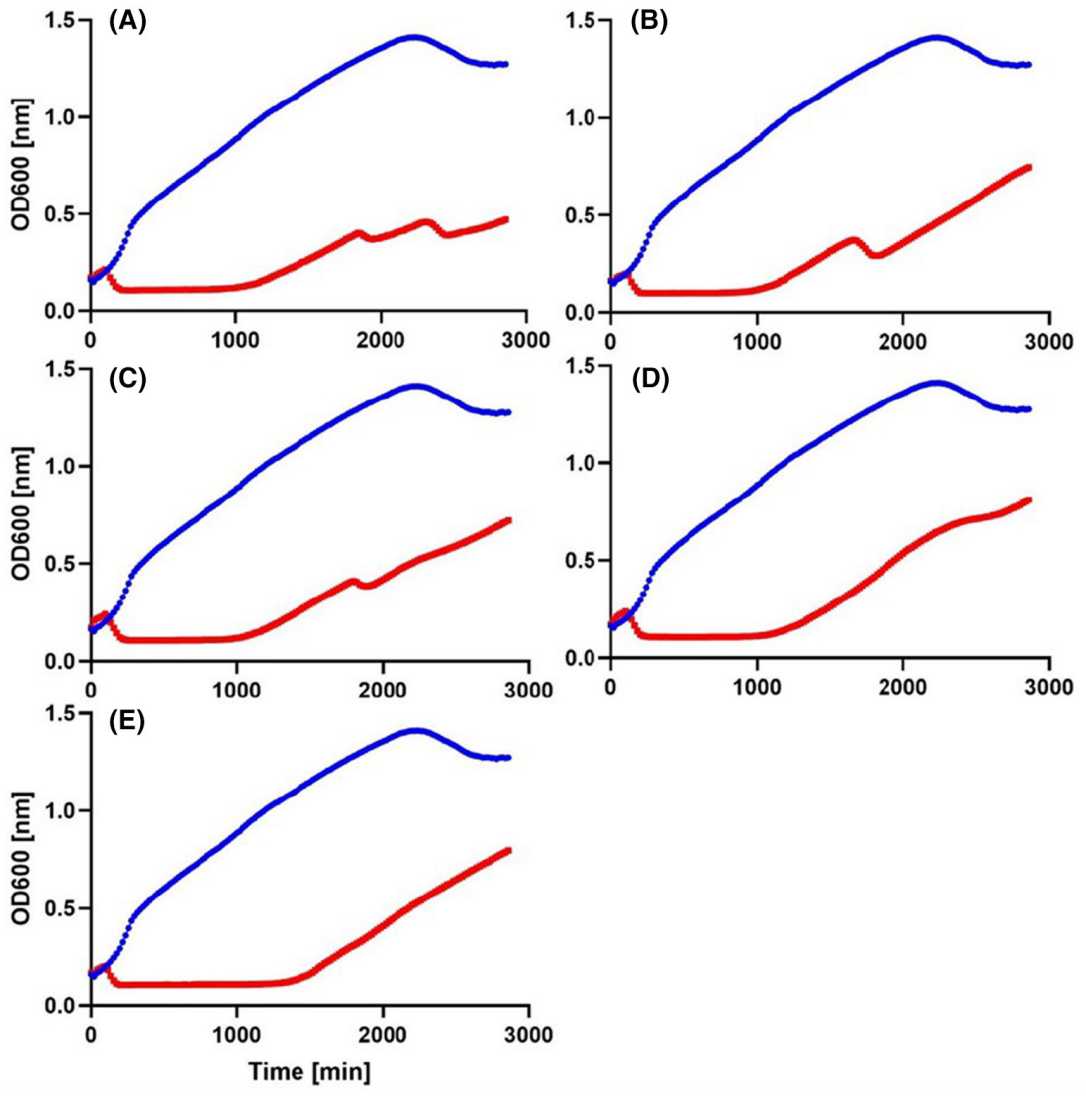
Arms race dynamics reduce in frequency when bacteria are exposed to more phage genotypes. Shown are in vitro killing curves of phage MR6 individually or in combination with MR1, MR4, MR14 and MR15 at multiplicity of infection of 0.01 on *Pseudomonas syringae* pv. *syringae* strain 9097 (*Pss*) with measurements taken over 3000 min (50 h). (A) phage MR6, (B) phage MR6 and MR15, (C) phage MR6, MR14 and MR15, (D) phage MR6, MR4, MR14 and MR15, (E) phage MR6, MR1, MR4, MR14 and MR15. Top blue line is *Pss* and bottom red line is *Pss* and phage(s). The experiment was repeated twice and each line represents the mean of three replicates. Note that the *Pss* line is the identical data set for each graph, though all treatments were carried out at the same time. Statistical analysis has been included in Table S2.

Of the 13 phage genomes examined in detail in Rabiey et al. (2020), each phage could be distinguished into one of five genomotypes based on their genome sequences: MR1 and MR2; MR4; MR5‐8, MR12, MR16, MR18; MR14; MR13 and MR15. Thus, a representative of each of the five genomotypes was chosen for this study as: MR1, MR4, MR6, MR14, MR15. The first aim was to study how each phage on its own, and as a cocktail of two, three, four or five phages, could reduce the density of *Pss* strain 9097 in vitro and to determine whether this leads to the emergence of phage‐resistant bacterial genotypes. Killing curve assays showed that all combinations exhibited the ability to inhibit bacterial growth over a 48‐h (3000 min) period compared to *Pss* only (Figure 1, Figure S2 and Table S2). Focussing on one phage as an exemplar, observation of the single phage killing curve for MR6 showed that bacterial resistance started to emerge after 16 h (1000 min), followed by two small drops in density, likely indicating an arms race between the phage and bacteria (Figure 1A). However, as the combination of phages used in the experimental treatments was increased from two to three, four and five (Figure 1B–E), these small density drops reduced in frequency and amplitude, and were completely absent when all five phages (cocktail 5, 5C) were used. Similar observations were made for most of the other single phage/multiple phage combinations (Figure S2A–L). These observations indicate that the bacterial host can adapt to become resistant to all five phages as well as phage mixtures and that a cocktail of five phages seems to prevent potential arms race dynamics.

### Bacterial phage resistance emerged over time

**FIGURE 2:**
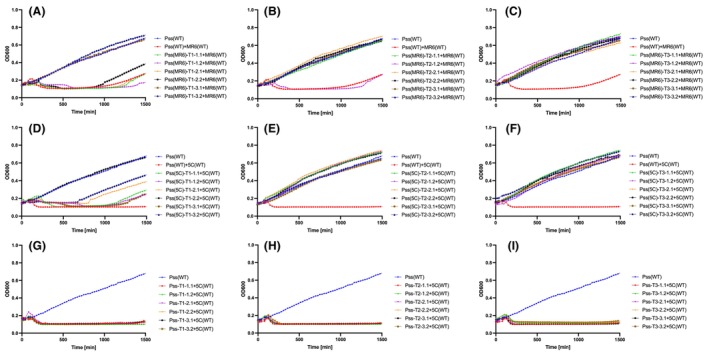
Phage‐resistant bacterial mutants dominate the bacterial population after phage application. Shown are in vitro killing curves of wild‐type phage MR6 and cocktail 5 (5C) at multiplicity of infection of 0.01 on wild‐type (WT) *Pseudomonas syringae* pv. *syringae* strain 9097 (*Pss*) and *Pss* phage‐resistant isolates collected at three time points (T1 [25 h]—A, D, G, T2 [47 h]—B, E, H, T3 [66 h]—C, F, I) over 25 h (1500 min). The 66‐h killing curve assay was done with *Pss* and phage MR6 (A, B, C), cocktail of 5 phages (5C) (D, E, F) and *Pss* alone (no phage treatment) (G, H, I) treated with wild‐type 5C. The experiment was repeated twice and each line represents the mean of two replicates. Note that the *Pss* (WT) line is the identical data set for each graph, though all treatments were carried out at the same time. Statistical analysis has been included in Table S2.

The killing curve assays in Figure 1 suggest that bacterial phage resistance in the population for the five‐phage mixture potentially occurs without fluctuations in resistance and counter‐resistance (bacteria and phage evolving to new genotypes in the population). To examine this in more detail, killing curves were repeated for each of the five phages individually and the combination of five phages (5C) for a period of 66 h (3960 min). This enabled an assessment of whether potential arms race or fluctuations occurred over a longer time period and enabled a sampling strategy that follows changes in phage killing/bacterial resistance. Bacterial samples were taken at T1 (25 h, when phage resistance started to emerge), at T2 (47 h, when phage resistance emergence was at its peak) and at T3 (66 h, at the end of the experiment). From each replicate (three) and time point, three bacterial isolates were collected (27 colonies in total per phage or 5C). A killing curve assay was then performed to check if the wild‐type phages (ancestral) were still able to infect the bacterial isolates collected at different time points during the course of the 66‐h experiment. For example, observation of the single phage killing curve for MR6 at T1 (25 h) showed that wild‐type MR6 could still infect the bacterial isolates (except from two isolates T1‐3.1 and ‐3.2) (Figure 2A–I and Table S2). All *Pss* isolates (except one, T2‐1.2) collected at T2 (47 h) and T3 (66 h) were resistant to the wild‐type MR6 (Figure 2B,C). This was similar across all other phages and 5C (Figure 2, Figure S3I–II and Table S2). This might suggest that enough mutations have already accumulated in *Pss* from T1, which were being selected from that point onward, therefore, leading to resistance to wild‐type phages.

### The evolution of phage resistance results in changes in pathogen fitness

**FIGURE 3:**
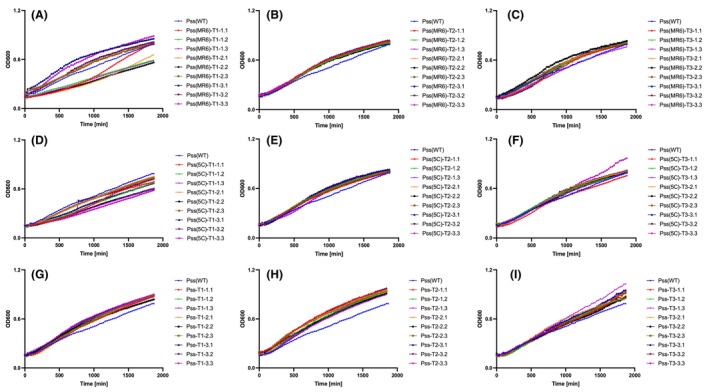
Phage‐resistance mutations in *Pss* 9097 initially reduce bacterial fitness in vitro before recovering over time. In vitro growth curves of *Pseudomonas syringae* pv. *syringae* strain 9097 (*Pss*) phage‐resistant isolates collected at three time points (T1—A, D, G, T2—B, E, H, T3—C, F, I), during a 66‐h killing curve assay with phage MR6 (A, B, C), cocktail of 5 phages (5C) (D, E, F) and *Pss* with no phage treatment (G, H, I). Each experiment was repeated twice and each line represents the mean of two replicates. Note that the *Pss* (WT) line is the identical data set for each graph, though all treatments were carried out at the same time. Statistical analysis has been included in Table S2.

The emergence of phage resistance may well result in fitness costs to the bacterial pathogen. This was tested by a range of in vitro and *in planta* assays. At T1 (25 h), the growth of phage‐treated isolates was significantly different from the control and the wild‐type strain (Figure 3A; A–I). By T2 (47 h) and T3 (66 h), the difference in growth was less pronounced and there was no difference between the growth curves of the phage‐treated and control isolates (Figure 3B,C). This pattern was repeated across all phage treatments (Figure S4A–L), though the differences between the growth curve at T1 for 5C were less pronounced compared to single phages (ANOVA, *p* = 0.01, Figure 3D). Together, these observations suggest that bacterial mutations for phage resistance incur a cost on bacterial fitness that is eventually overcome in time, presumably by compensatory mutations.

The emergence of phage resistance may result in a cost on either the pathogen's ability to cause disease (pathogenicity) or the virulence (severity) towards the host plant, cherry. Phage‐resistant isolates were inoculated on detached cherry leaves by infiltration and the bacterial populations were enumerated over time. All isolates from the phage‐free control treatment caused disease symptoms (Figure S5A–H) and grew to consistently high levels within the leaf (Figure S6A). Isolates from the MR1 and MR15 treatments showed no changes compared to wild‐type isolates, though there was a larger variation in growth for some isolates. Five isolates from the MR4, MR6 and MR14 treatments were observed to have lost pathogenicity and did not grow *in planta* (exemplified by the MR6‐treatment graph, Figure S6C, T1‐2.1, T1‐3.1 and T3.2.1), indicating some mutants did experience severe fitness defects from the mutations conferring phage resistance. Interestingly, the 5C isolates recovered from T2 and T3 exhibited a large degree of growth variation (Figure S6F) and two isolates (T2‐3.1 and T3‐2.1) were unable to grow *in planta* (Figure S6A–G). These observations suggest that there is no apparent fitness cost to the pathogenicity and virulence in some phage‐resistant isolates under the conditions tested, while others are compromised.

### Both phages and bacteria evolve resistance‐breaking innovations

**FIGURE 4:**
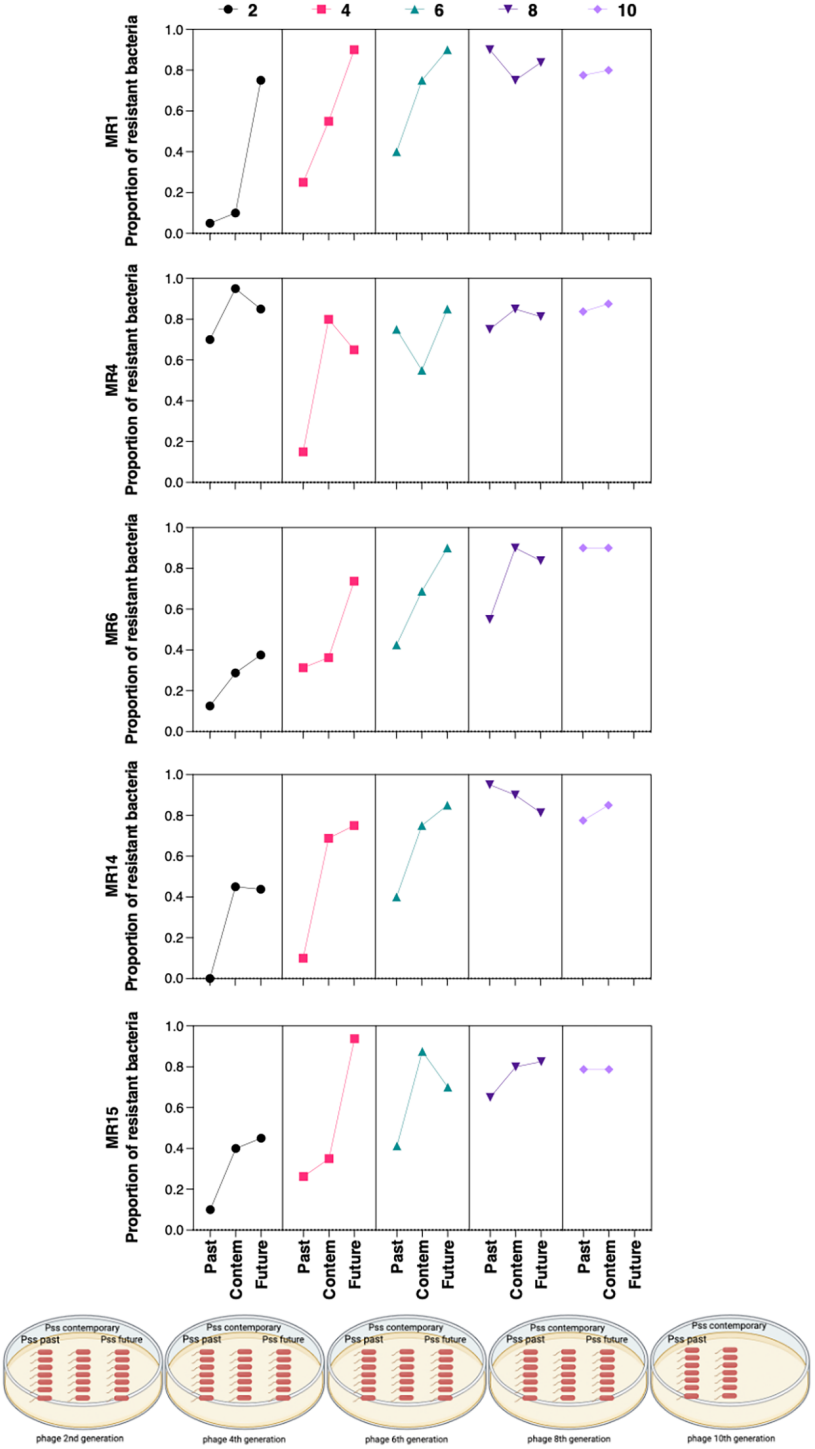
The proportion of resistant bacteria increases over time when bacteria are coevolved with phage. The proportion of *Pseudomonas syringae* pv. *syringae* strain 9097 resistance to phage MR1, MR4, MR6, MR14 and MR15 was tested over time. The experimental coevolution was done by inoculating 6‐mL King's medium B (KB) broth with phage and bacteria. After 48‐h incubation at 27°C, both bacteria and phage were recovered and transferred to new KB broths. This was repeated for 10 transfers with sample population collections every second transfer. Each phage, collected at each coevolution generation, was tested against the bacterial populations to past (two transfers previous), present (contemporary) and future (two transfers subsequent). Statistical analysis has been included in Table S2.

One key attribute for the use of phages in a therapy to treat bacterial disease is to ensure that the treatment is robust, that is, the use of phages does not break down due to the emergence of bacterial phage‐resistant mutants. The observations above indicate that bacteria treated with both single phages and a five‐phage cocktail can, in fact, result in the emergence of phage‐resistant bacterial mutants. Moreover, some of these mutants did not appear to experience major fitness deficits *in planta*. This might suggest that phage evolution to infect the mutants over time is limited and thus that bacterial phage‐resistant mutants will persist. To test this, an experimental evolution approach was employed to examine the patterns of coevolution between the five single phages and 5C with *Pss*, by passaging for 10 transfers, that is, *Pss* and one phage were co‐incubated together for 48 h, an aliquot was removed to inoculate a fresh broth and also to allow the bacteria and phage to be recovered. The proportion of bacteria from each passage that had developed resistance to the phage was determined by phage–bacteria streak plates to look for loss of growth where the bacterial line crossed the phage line; this was done for the immediate ancestral, contemporary and future bacterial strains over the contemporary phage generation. Over the course of the experiment, less bacterial phage resistance was observed in the ‘past bacteria‐future phages’ and ‘contemporary bacteria‐contemporary phages’ than in the ‘future bacteria‐past phages’. Bacteria from the future were more resistant to the phage from the past than to their contemporary phages or phages from the future. Notably, bacterial resistance increased in frequency over the 10 passages, though 100% resistance was not observed during this period. This indicates that although phage resistance emerges and increases in the bacterial population, phages were still able to infect and reduce the density (Figure 4). When phages collected at the last transfer were tested against the contemporary bacteria (bacteria collected at transfer 10) via spot assay, the production of plaques was observed, indicating the presence of infective phages present in the culture. This suggests that despite the high level of bacterial resistance at P10, some infective phages remain.

**FIGURE 5:**
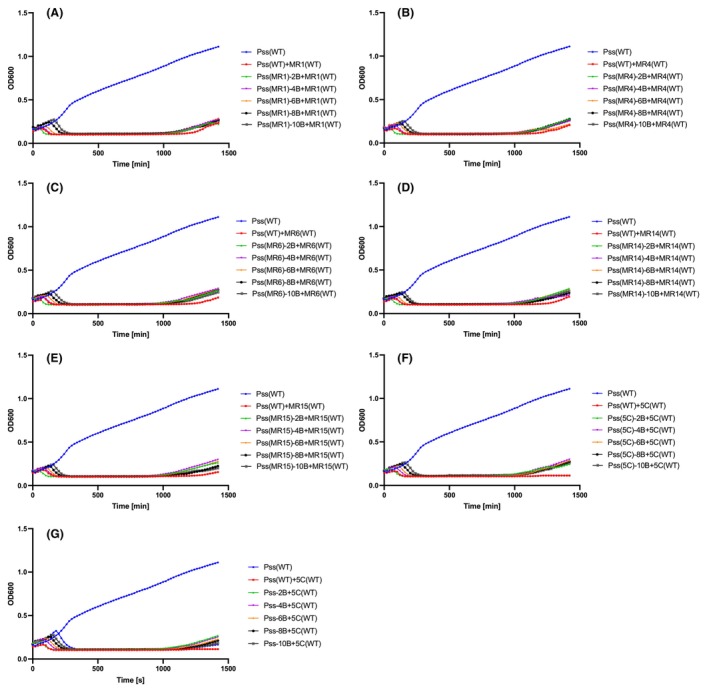
Coevolved *Pss* strains remain susceptible to wild‐type phages and the five‐phage cocktail 5C. In vitro killing curves of phages MR1 (A), MR4 (B), MR6 (C), MR14 (D), MR15 (E), 5C (cocktail of five phages, F) and ancestral *Pss* passaged without phage treated with cocktail 5C (G) at multiplicity of infection of 0.01 on wild‐type (WT) *Pseudomonas syringae* pv. *syringae* strain 9097 (*Pss*) and *Pss* phage‐coevolved isolates collected at 2nd (2B), 4th (4B), 6th (6B), 8th (8B) and 10th (10B) transfer, during an experimental coevolution with *Pss* and phage MR1, MR4, MR6, MR14, MR15 and 5C. The experiment was repeated twice and each line represents the mean of two replicates. Note that the *Pss* (WT) line is the identical data set for each graph, though all treatments were carried out at the same time. Statistical analysis has been included in Table S2.

To investigate if the ancestral wild‐type phages were still able to infect the coevolved *Pss* isolates collected at different transfer times, a killing curve assay was performed. Wild‐type phage MR6 and 5C were still able to kill the coevolved *Pss* (Figure 5A–G), this was similar across all phages. This suggests that the mutations in *Pss*‐phage resistance isolates were not sufficient to prevent the wild‐type phage infection and even when phage resistance emerges in the population, wild‐type phages are still able to infect them.

**FIGURE 6:**
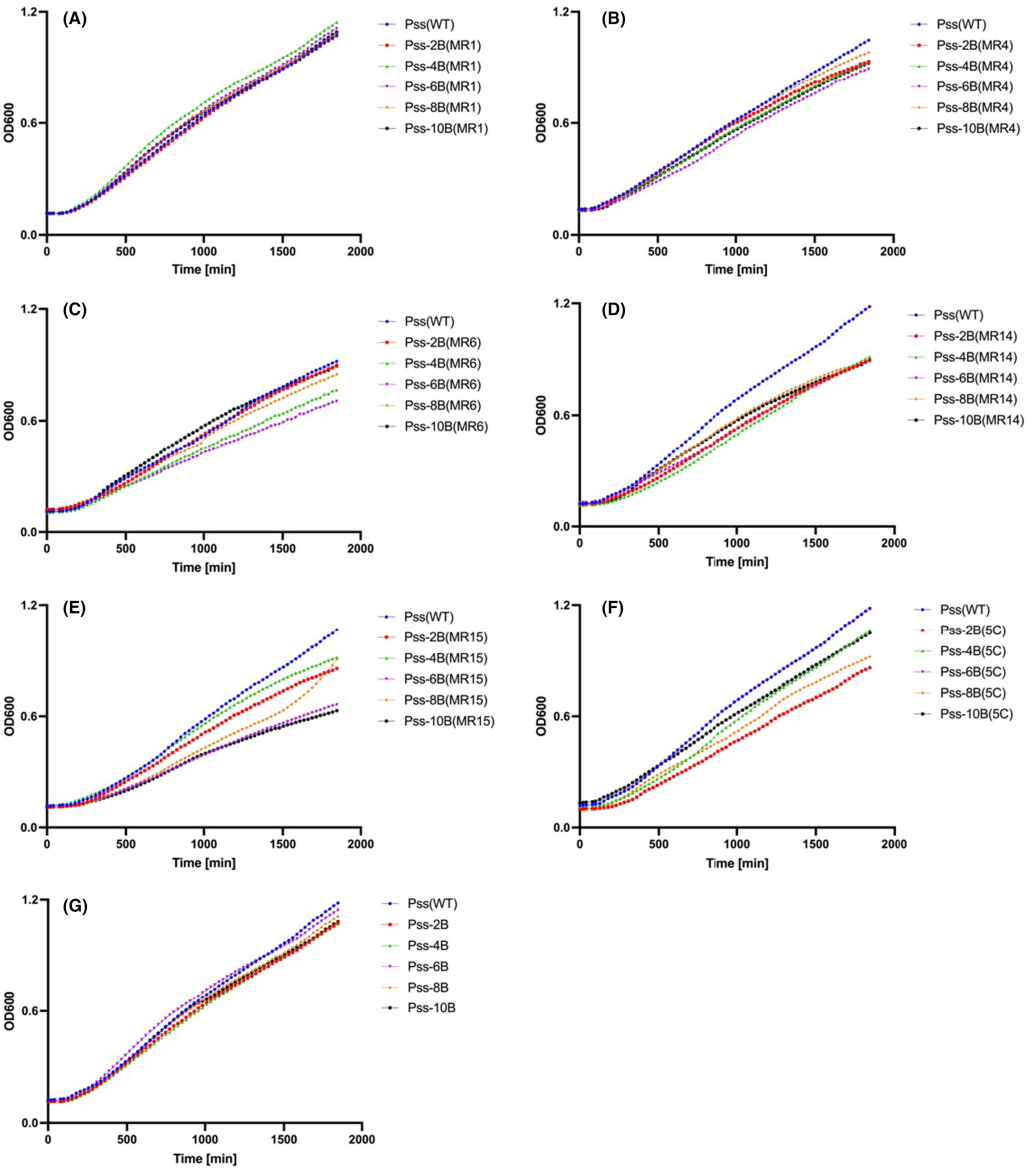
Coevolved *Pss* strains exhibited either a similar or slower growth pattern compared to the ancestral *Pss*. In vitro growth curve of coevolved generations (2B to 10B) of *Pseudomonas syringae* pv. *syringae* strain 9097 (*Pss*) after coevolution with phage MR1 (A), MR4 (B), MR6 (C), MR14 (D), MR15 (E), 5C (cocktail of five phages [5C], F) and *Pss* passaged with no phage treatment (G) treated with cocktail 5C. *Pss* phage‐coevolved isolates were collected at 2nd (2B), 4th (4B), 6th (6B), 8th (8B) and 10th (10B) transfer. The experiment was repeated twice and each line represents the mean of two replicates. Note that the *Pss* (WT) line is the identical data set for each graph, though all treatments were carried out at the same time. Statistical analysis has been included in Table S2.

One possible impact of the mutations in the resistant strains could be an effect on bacterial functioning that can impact fitness, reflected in altered growth rates. The fitness costs of resistance on growth rates of coevolved bacteria after coevolution with each phage were compared with the ancestral *Pss* via growth curve assays. All *Pss* generations, collected from each *Pss*‐phage coevolution, from every other transfer, exhibited either a similar or slower growth pattern to the ancestral *Pss* (Figure 6), similar to the findings of the 66‐h experiment (Figure 2). It was observed that there were some subtle increases and decreases in growth through the passages (2B being lower, going through increase and decrease in fitness and resulting in 10B being most similar to WT). Generation 2B to 8B of *Pss* coevolved with MR6 grew slower than the ancestral *Pss*; however, generation 10B had similar growth. *Pss* coevolved with 5C, MR14 and MR15 grew slower than the ancestral *Pss* (Figure 6A–G). This indicates that the mutation of phage resistance in coevolved *Pss* strains impacts fitness at different periods, but that fitness can increase over time towards wild‐type levels.

These isolates were investigated for the cost of phage resistance on the pathogenicity and virulence of phage coevolved isolates via the detached cherry leaf assay and the bacterial population was enumerated and disease symptoms scored. *Pss* coevolved with phage MR6 and cocktail 5 was still able to cause symptoms (Figure S7A–G) and grow on cherry leaves (Figure S8). Therefore, no obvious fitness costs were associated with *Pss*‐MR phage coevolution.

### Whole‐genome sequencing of mutants reveals *Pss* genes essential for phage infection and lipopolysaccharide might be a key receptor for MR phages

To identify bacterial genes with mutations that may be involved in phage resistance, whole‐genome sequencing and variant calling analysis were performed on colonies collected for the 66‐h phage–bacteria killing curve assays at T1, T2 and T3 and also selected bacterial mutants from the coevolution experiment (transfer 2B to 10B), for the five individual phages and cocktail 5C.

**FIGURE 7:**
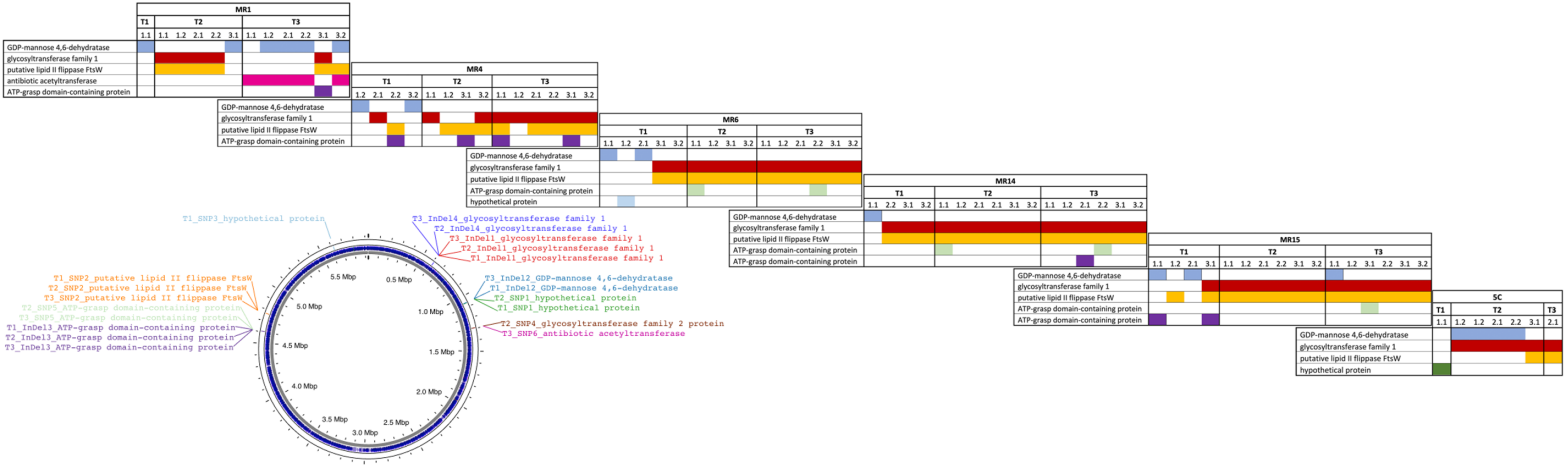
Mutations identified in *Pseudomonas syringae* pv. *syringae* strain 9097 (*Pss*) after single and phage cocktail treatments. Colonies were collected three times (T1, T2 and T3) during the course of 66‐h killing curve assay, with MR1, MR4, MR6, MR14, MR15 and cocktail of five phages (5C). Six colonies were whole genome sequenced at each time point (e.g. T1‐1.1, ‐1.2, ‐2.1, ‐2.2, ‐3.1 and ‐3.2) and variant calling was employed (full details in Table S1). *Pss* Lipopolysaccharide gene cluster is shown in Figure S8B.

For the phage–bacteria 66‐h killing curve assay, three colonies per time point (T1, T2 and T3, nine colonies in total) per phage–bacteria interaction were sequenced. All SNPs are described in Figure 7 and Table S3. In total, four InDels and six SNPs were identified, and mutations in genes for glycosyltransferase family 1 protein (*gst1*, InDel2), GDP‐mannose 4,6‐dehydratase (*gmd*, InDel3) and FtsW protein (*ftsW*, SNP5) were the most frequently mutated genes during *Pss*–phage interaction, though mutants altered in an ATP‐grasp domain‐containing protein (*agd*) were also observed in some strains for all of the single phage treatments. Notably, a mutation in *gmd* was observed in T1 strains for all five individual phages, but not until T2 for 5C. The *gmd* mutation persists in the population treated with MR1, but is identified less frequently in T2 and T3 colonies from other phage treatments. Conversely, the *gst1* and *ftsW* mutations were not identified in T1 for MR1, but did occur in T2 and T3, while for the other phage treatments, *gst1* and *ftsW* emerged in T1 and were predominant in T2 and T3.

**FIGURE 8:**
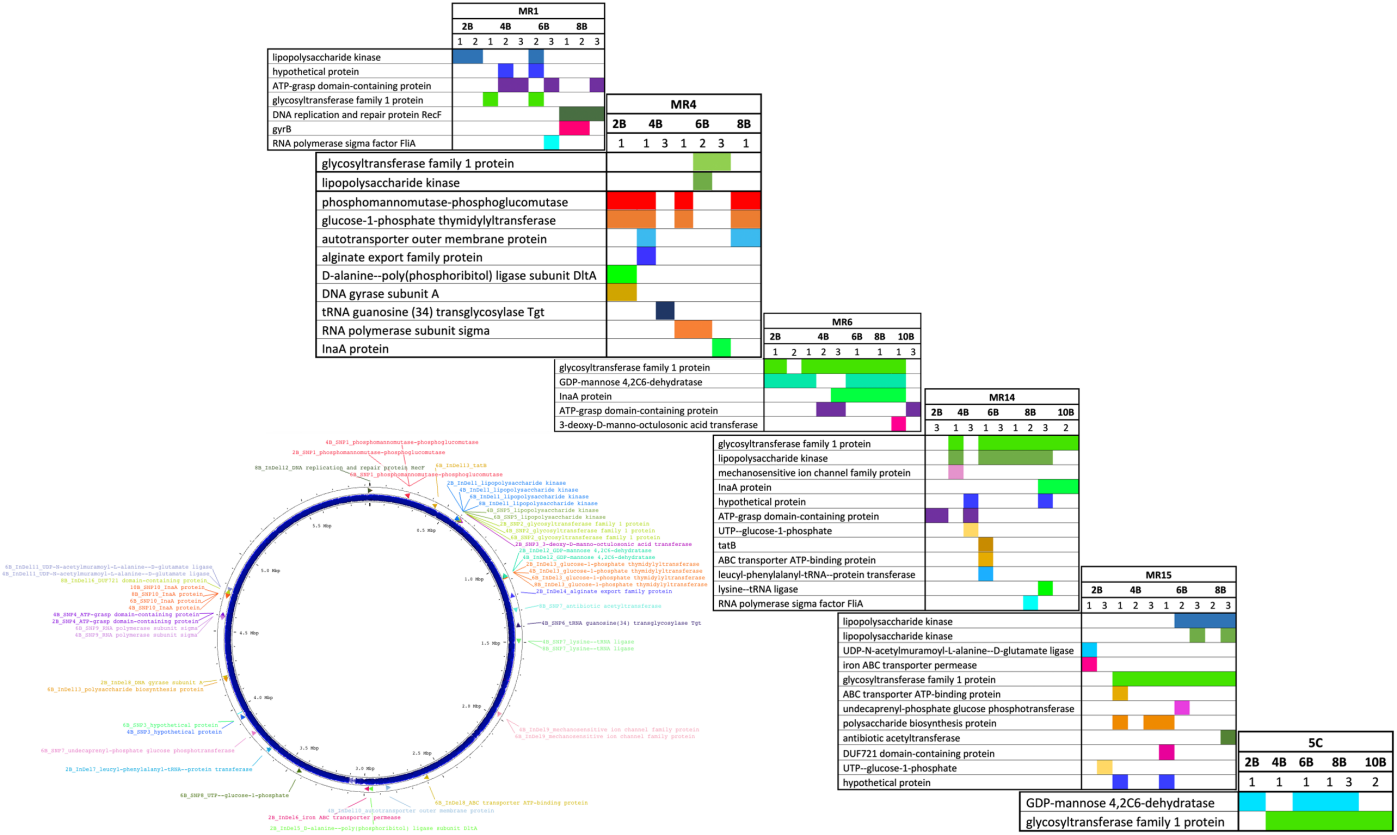
Mutations identified in *Pseudomonas syringae* pv. *syringae* strain 9097 (*Pss*) after single and phage cocktail coevolution. Colonies were collected at generation 2B, 4B, 6B, 8B and 10B during the experimental coevolution of *Pss* with phage MR1, MR4, MR6, MR14, MR15 and cocktail of five phages (5C). Three colonies were whole genome sequenced at each generation and variant calling was employed. The *Pss* Lipopolysaccharide gene cluster is shown in Figure S8B.

For the coevolution experiment, three colonies were sequenced from every second generation of *Pss* coevolved with phages MR1, MR4, MR6, MR14 and MR15 and 5C. In total, 16 InDels and 15 SNPS were identified. All InDels and SNPs are described in Figure 8 and Table S4. Some of the same mutations were observed as seen in the 66‐h killing curve experiment, that is, *gst1* (SNP5, *Pss*‐MR1, ‐MR6, ‐MR14, ‐MR15 and ‐5C), *gmd* (InDel4, *Pss*‐MR6 and 5C) and *agd* (SNP14, Pss‐MR1, ‐MR6 and ‐MR14), with mutation in an *inaA* (ice‐nucleation) (SNP15, Pss‐4, ‐MR6 and ‐MR14) also frequently occurring during *Pss*‐phage coevolution. Notably, the *ftsW* mutation was not observed in any of the mutants.

Interestingly, different mutations were most frequently found in different phage treatments in the coevolutionary experiment. For the *Pss*‐MR4 treatment, mutations in phosphomannomutase/phosphoglucomutase (*pmm*, SNP2) and glucose‐1‐phosphate thymidylyltransferase (*gpt*, InDel5) were predominant, whilst mutations in a lipopolysaccharide kinase (*lpk*, InDel3) were predominant in the *Pss*‐MR14 and *Pss*‐MR15. Remarkably, only two predominant mutations in *gmd* and *gst1* were observed for the cocktail 5C indicating that the use of all five phages reduces the spectrum of mutations that occur in the bacteria, but also that the bacterium can effectively overcome a complex mixture of five phages.

Some of the mutated genes are essential for biosynthesis of lipopolysaccharide (LPS) and are critical for the integrity of the bacterium's cell wall. For example, *gst1*, *gpt*, *lpk* and *gmd* are involved in the biosynthesis of LPS core oligosaccharide, LPS core phosphorylation and the biosynthesis of LPS O‐antigen.

**FIGURE 9:**
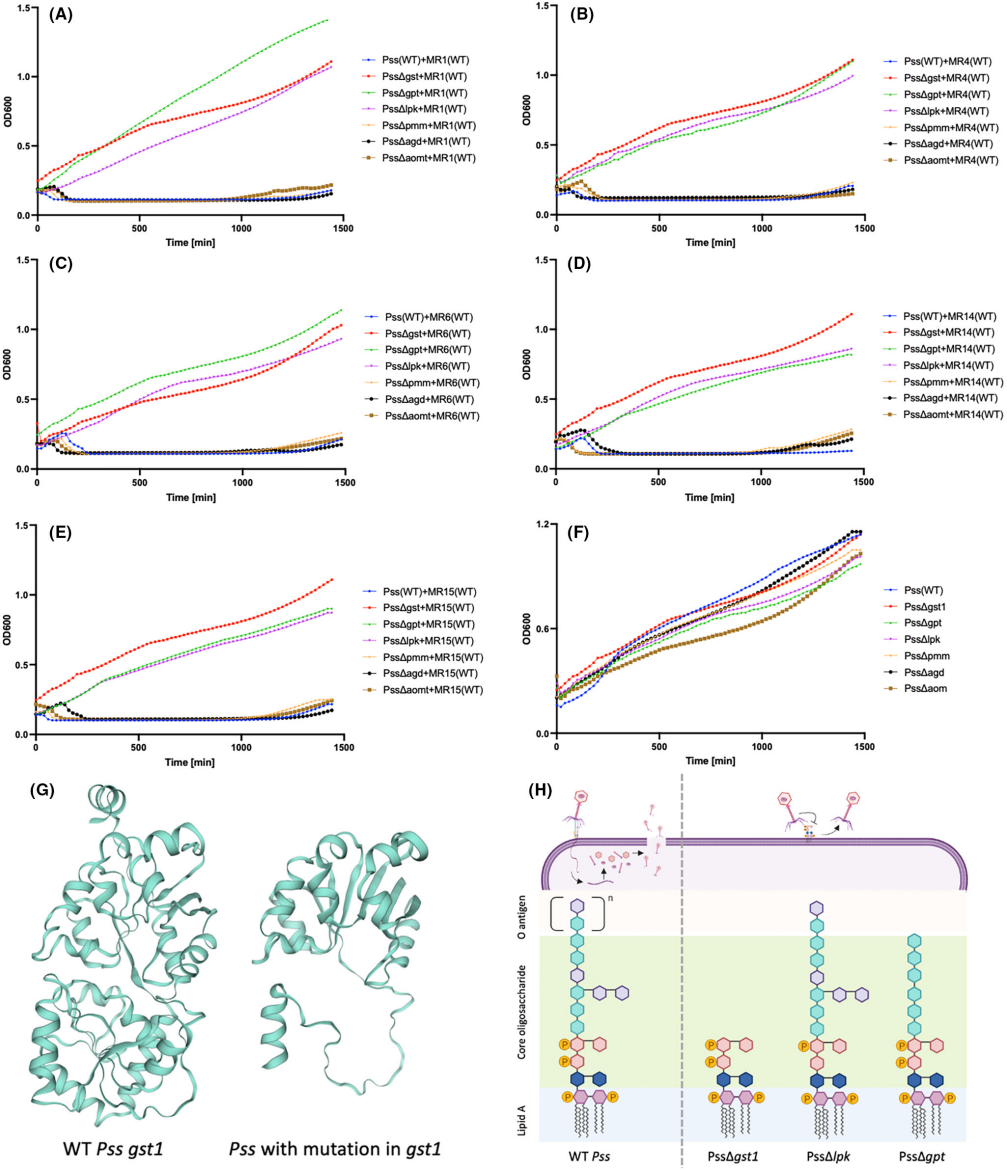
Deletion of genes involved in LPS synthesis prevents wild‐type phages and 5C from killing the bacteria. An in vitro killing curve of phages MR1 (A), MR4 (B), MR6 (C), MR14 (D) and MR15 (E) on *Pseudomonas syringae* pv. *syringae* strain 9097 (*Pss*) wild type (WT) and glycosyltransferase family 1 (*gst1*), glucose‐1‐phosphate thymidylyltransferase (*gpt*), lipopolysaccharide kinase (*lpk*), phosphomannomutase (*pmm*), ATP‐grasp domain‐containing protein (*agd*), autotransporter outer membrane protein (*aom*) mutants (Δ). *Pss* WT and all *Pss* mutants with no phage are shown in (F). The predicted native protein structure (G) of *gst1* in *Pss* WT and the predicted protein structure of *gst1* (BKC06_002880) how mutation identified in *gst1* changes the structure (created in Swissmodel.expasy.org). The last panel (H) is the putative structure of lipopolysaccharide (LPS) in *Pss*, consisting of lipid A, core and O antigen and hypotheses on how mutation in proteins involved in LPS biosynthesis can lead to phage resistance (created in Biorender.com). The experiment was repeated twice and each line represents the mean of two replicates. Note that the *Pss* (WT) line is the identical data set for each graph, though all treatments were carried out at the same time. Statistical analysis has been included in Table S2.

Based on these observations, LPS core and O‐antigen were hypothesized as being an important target receptor for the phages, probably for adhesion. Therefore, deletions of *gst1*, *gpt* and *lpk* in the *Pss* 9097 genome were made to test this hypothesis. Gene deletions of three other genes that were identified in the phage–bacteria coevolution experiments were also made: *agd*, *pmm* and an autotransporter outer membrane protein (*aom*). The impact of each deletion on phage–bacteria interaction were then observed by killing curve assays. Wild‐type *Pss* and *Pss* mutants were grown with the five respective phages. Wild‐type phages reduced the wild‐type *Pss* density almost immediately, but they were not able to reduce the *Pss*Δ*gst1*, *Pss*Δ*lpk* and *Pss*Δ*gpt* mutant densities. However, all wild‐type phages were able to reduce the *Pss*Δ*agd*, *Pss*Δ*aom* and *Pss*Δ*pmm* mutant densities (Figure 9A–H) and it was notable that an increase in bacterial density, presumably due to the emergence of phage‐resistant mutants, occurred at around 20 h similar to the observation with wild‐type *Pss* with the phages. Deletion of all the genes, except *gst1*, gave small but significant reductions in the growth of *Pss*, compared to the wild type (Figure 9F and Table S2). These were most prominent for *agd* and *aom*, with *lpk* and *gpt* mutations impacting growth in the later time points. Considering, *gst1* and *lpk* are within the lipopolysaccharide gene cluster, and *gpt* contributes to the synthesis of *O‐*antigen, it would appear that lipopolysaccharide is the key receptor for MR phage adsorption before infection. Indeed, comparison of the predicted protein structure of *gst1* for the mutant versus the wild type indicates that the protein is likely truncated and missing the C terminus (Figure 9G), thus likely preventing correct synthesis of core oligosaccharide and O‐antigen (Figure 9H).

### Rational cocktail design by including evolved phages overcame bacterial resistance

**FIGURE 10:**
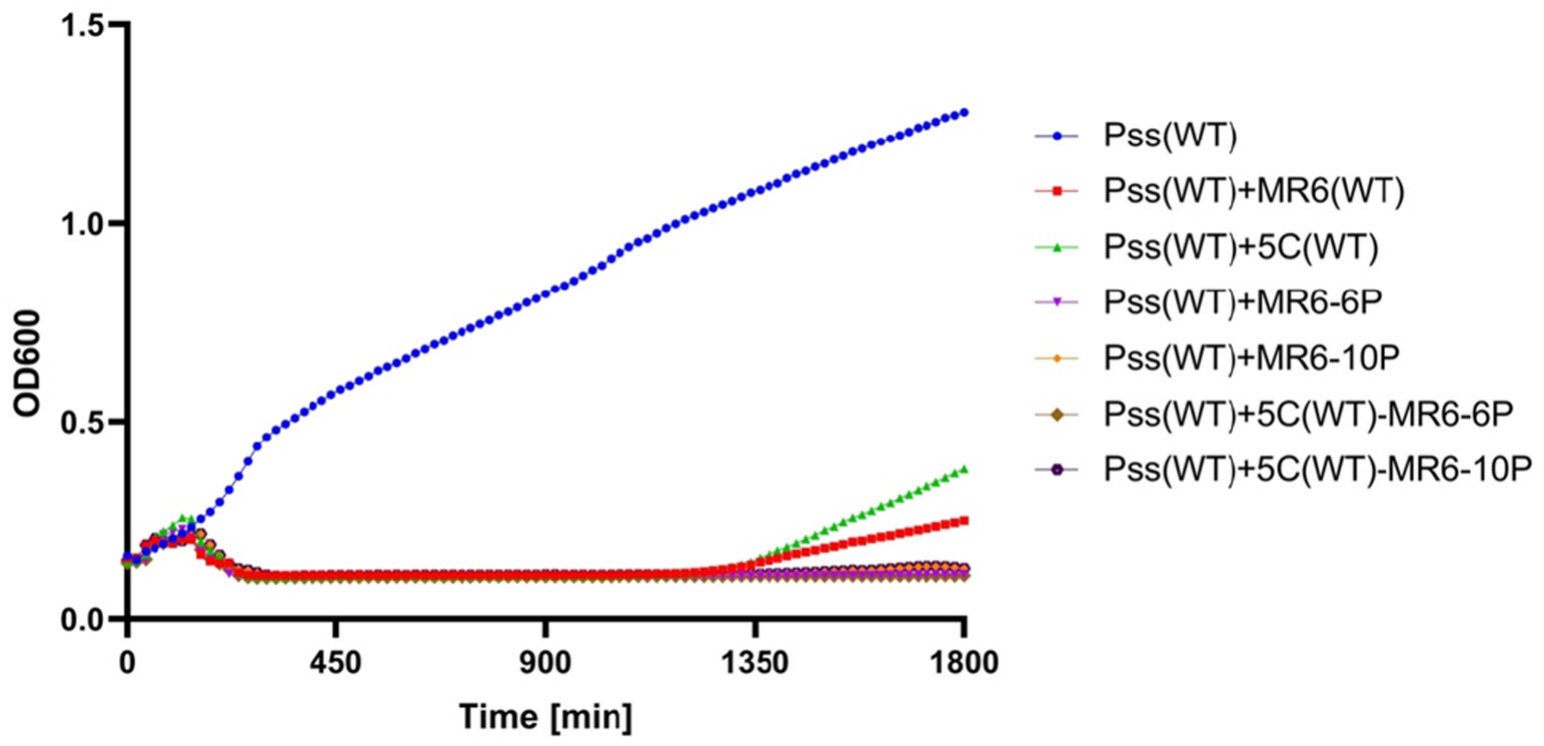
The addition of trained phages to cocktail 5C reduces the emergence of phage resistance. An in vitro Killing curve of phage MR6, cocktail (5C) and MR6 coevolved generation 6 (6P) and 10 (10P) on *Pseudomonas syringae* pv. *syringae* strain 9097 (*Pss*). The experiment was repeated twice and each line represents two replicates. Note that the *Pss* (WT) line is the identical data set for each graph, though all treatments were carried out at the same time.

In the phage–bacterium coevolutionary experiment, it was observed that phages collected at the later coevolved generations had become very efficient at killing the wild‐type *Pss*, presumably retaining the ability to attach to the wild‐type cells as well as the evolved mutants. For example, coevolved phage MR6 from generation 6P and 10P reduced the wild‐type *Pss* density so efficiently that no bacterial growth was observed above OD 0.25, with no bacterial resistance emerging during 2000 min (25 h, Figure 10). Given these phages are likely countering the two dominant mutations, in *gst1* and *gmd*, both phages were added to the cocktail 5C, to understand if this modified cocktail could reduce bacterial resistance emergence. No bacterial resistance was observed compared to when wild‐type 5C was applied. This suggests that phages can be trained through coevolution to generate more effective phages that are, in essence, ready to infect a bacterial mutant emerging during the phage–bacteria interaction.

## DISCUSSION

Phages have been used in clinical applications for many years and have become useful treatments for controlling bacterial disease in plants. Our previous work studying phages that can attack cherry canker pathogens found a set of phages with diverse genomes that showed promise for control of the disease (Rabiey et al., 2020). However, bacterial growth dynamics in vitro suggested that they could overcome phage treatment, probably via phage‐resistant mutations arising. Although in vitro observations using a nutrient‐rich medium are not representative of the natural environment, this does highlight that phage resistance could potentially emerge and thus the use of phages might drive bacterial pathogen evolution in undesirable ways. Here, a detailed analysis of the dynamics of in vitro phage–bacteria interaction and coevolution is presented using the cherry pathogen (*Pss*) and five different phage genomotypes. First, the phage efficacy in reducing bacterial densities in vitro was measured, with individual phages and when mixed together in a combination of two‐ to five‐phage mixtures. The more phages that were added to the mix, the quicker phage resistance emerged, suggesting that phages might be competing with each other for attachment sites to the bacterial cell surface, reducing their efficacy. To investigate this, MR phage–*Pss* interactions were followed by killing curve assays performed over a 66‐h period and coevolution over 10 transfers to examine the emergence of phage resistance throughout both experiments.

As phage resistance becomes prevalent within bacterial populations, the mechanisms employed to avoid phage invasion often lead to a trade‐off in other crucial traits associated with the fitness of the bacteria, such as growth rates and pathogenic potential. For both the in vitro MR phage‐*Pss* interaction and the MR phage‐*Pss* coevolution experiments, the in vitro growth of *Pss* mutants was only slightly impaired. However, the development of phage resistance did not affect the pathogenicity of most strains, though for some, there was a complete to partial loss of pathogenicity in cherry leaves, from strains derived from the 66‐h killing curve experiment. Our genome sequence analysis did not indicate any obvious genomic differences between the mutants to explain these phenotypic changes, but might reflect some kind of alternative change (e.g. gene inversion, methylation) that our analysis did not detect. Our finding is partially in contrast to the work of Hernandez and Koskella (2019) who made a key observation that the development of phage resistance in *P*. *syringae* pv. *tomato* PT23 impaired pathogenicity on tomato plants. This might indicate a decreased likelihood of phage‐resistant mutants emerging and surviving to high population levels in plants, helping to reduce pathogen levels and disease spread and symptoms. However, our observations match those of Warring et al. (2022) which found that *P*. *syringae* pv. *actinidae*, the pathogen of kiwifruit, treated with ΦPsa374 developed phage resistance and only exhibited impaired growth in vitro, but were still pathogenic on kiwifruits. Why this variation occurs is unclear, perhaps pointing to the experimental design, and phage application and selective environment in vitro versus *in planta*. It does, however, raise the importance of analysing the outcomes of phage resistance on pathogen performance in its host as well as understanding the effectiveness of the phages being applied and whether modifications to the treatment approach are needed. This would guide treatment design towards using the phages in a cocktail with phages that target different receptors, to both reduce the emergence of phage resistance and reduce bacterial pathogenicity/virulence. Moreover, the magnitude of costs associated with developing resistance is likely to vary depending on the mechanism of phage resistance that occurs (Refardt & Kümmerli, 2013) and the environment in which mutants are selected for. Thus, bacterial mutations to phages that attach to different targets are much more likely to impact bacterial fitness and survival and thus be more effective. Alternatively, a design that uses phages evolved to adsorb to adhesion molecules that will mutate when challenged by wild‐type phage may also be effective.

As bacteria develop resistance, phages undergo their own evolutionary adaptations to counter this resistance. An optimal scenario would involve these evolved phages becoming more proficient at infecting their bacterial hosts. James et al. (2020) found that the proportion of *Pseudomonas* bacteria resistant to future generations of coevolved phage decreased with later generations. They found that coevolution with a different phage saw a different pattern with some past bacteria more resistant to future coevolved phages. Storey et al. (2020) also saw a similar trend with variability in the resistance of *Pseudomonas* bacteria to future phages. One very promising observation from our study was that coevolved phages were more effective against wild‐type *Pss* than the original (ancestral) phages and that they appeared to reduce or prevent the emergence of resistance. Laanto et al. (2017) found that *Flavobacterium columnare* was susceptible to phages isolated years after its initial cultivation, but resistant to phages isolated prior to its isolation, indicating phage adaptation over time to break down phage resistance. This, therefore, highlights the potential approach for developing coevolved phages as a means to control current bacterial pathogens (Laanto et al., 2017), though ideally, phage genotypes would be screened through a wide range of bacterial isolates to determine if this trait was robust.

Based on the findings of others (Garbe et al., 2011; Li et al., 2019; Rabiey et al., 2020; Warring et al., 2022) and that the *Pss* genome encodes genes for LPS biosynthesis, this was predicted as a likely phage adhesion (and thus, mutation) target for at least one or more of the MR phages (Kutschera et al., 2019; Oechslin, 2018). In *E. coli*, a glycosyltransferase enzyme, encoded by the *waaG* gene, attaches the outer core hexose residuals to the Hep units (inner core), while a LPS kinase, encoded by *waaP*, modifies the first Hep residual of the inner core by adding a phosphate group (Valvano, 2015). Glycosyltransferase is also involved in the biosynthesis of core oligosaccharide in *Bordetella pertussis* (Geurtsen et al., 2009). In *Pseudomonas aeruginosa*, the *rmlA* gene encodes a putative glucose‐1‐phosphate thymidyltransferase predicted to be involved in the biosynthesis of dTDP‐L‐Rha, the core precursor of L‐Rha, which is later added to the LPS outer core and O‐antigen (Rahim et al., 2000). According to Wang et al. (2021) and Petitjean et al. (2021), deleting these genes leads to the synthesis of LPS without an outer core (*waaG*), LPS with an intact inner core but lacking the phosphorylation in HepI (*waaP*), or to changes in the LPS serotype as a consequence of modifications in the O‐antigen assembly (*rlmA*) (Wang et al., 2015). However, several mutations were located in putative LPS biosynthesis protein genes, such as *gst1* (glycosyltransferase family 1), *lpk* (lipopolysaccharide kinase) and *gpt* (glucose‐1‐phosphate thymidylyltransferase). Given that the predicted function of *gst1*, *lpk* and *gpt* is similar to *waaG*, *waaP* and *rlmA*, respectively, mutation of these genes likely leads to the synthesis of a modified LPS lacking the outer core region, L‐Rha residuals in the outer core or O‐antigen regions, or with non‐phosphorylated Hep residuals. This has been observed for other *Pseudomonas* phages (Olszak et al., 2019). Warring et al. (2022) showed that ΦPsa374 phage resistance in *P. syringae*. pv. *actinidiae* (*Psa*) arose in vitro and *in planta* through mutations in a glycosyltransferase (IYO_025560 in *Psa*) involved in LPS synthesis. Comparison of our *gst1* gene (BKC06_002880) indicated that this gene was different to the *Psa* locus (which matches BKC06_002885), but is in the same gene cluster (Figure S9A,B) and thus reinforcing the importance of LPS as a target for *P*. *syringae* phages. Using soil samples from around the base of cherry trees, no phages have been found yet that infect the mutants supporting the notion that LPS is one of the main receptor targets that *Pss* phages have evolved to interact with.

One notable observation was the effect of using single phages versus cocktail 5C on genomic changes within the pathogen populations. In the 66‐h continuous *Pss*–phage interaction experiments, *gst1* was the predominant mutation after treatment with MR4, MR6, MR14, MR15 and 5C, while *gst1* was initially dominant before *gmd* became dominant in the MR1‐treated population. Curiously, the an *ftsW* mutation emerged rapidly and fixed in many of the single phage treatment isolates, only emerging in the 5C treated population in two strains in T2 and T3. FtsW is part of the divisome complex involved in bacterial cell replication and is integral for peptidoglycan synthesis and assembly and construction of the cell wall during cell replication (Nguyen et al., 2023). Bacteriophage SP01 targets FtsL and disrupts FtsW recruitment in *Bacillus subtilis* (Bhambhani et al., 2020), thus there may be a similar MR‐phage impact on *Pss* cellular functioning and subsequent fine‐tuning to compensate. However, there appears to be a co‐linkage of *ftsW* mutations arising with the *gst1* mutation, but *ftsW* mutations do not emerge in the coevolution experiment. This suggests that the *ftsW* mutation is either a compensatory mutation or a stress‐response mutation observed in this particular experiment rather than a co‐requirement for phage resistance (Bhambhani et al., 2020). Another common mutation was in an ATP grasp‐domain protein emerging at time points in all single phage interactions, but not with the 5C treatment. ATP‐grasp enzymes are widespread in bacteria and also found in some phages (Fawaz et al., 2011; Iyer et al., 2021). They are often associated with a range of metabolic processes but have also been predicted to participate in phage‐resistance mechanisms (Anantharaman et al., 2012; Burroughs et al., 2015). The genomic context of the ATP‐grasp domain protein in *Pss* 9097 indicates that it is part of a metabolism‐ABC transporter operon. Neighbouring genes including a DUF89 domain‐containing protein gene and 16S rRNA pseudouridine (516) synthase protein gene predicted to participate in metabolite damage control mechanisms, including detoxification of excess or damaged phosphometabolites (Huang et al., 2016), and streptomycin resistance (Abedeera et al., 2023). Thus, it is not immediately obvious that the ATP‐grasp domain gene is involved in phage resistance, although it is localized in a potential genomic island of genes related with bacterial survival. This might, therefore, point to the mutations being compensatory or stress related rather than related to phage resistance, reinforced by the observation that ATP‐grasp domain gene mutations were infrequent in the coevolution experiment.

Most of the mutations occurring in the coevolution experiment appeared to be random, though there appeared to be some correlations between gene mutations and impact on fitness (growth): for example, *pmm* and *gpt*; *gst1* and *lpk*; *gmd* but not *gst1*. A striking observation from this experiment was that single phage use led to a much wider range of non‐LPS mutations compared to the population challenged with cocktail 5C, in which only two mutations in *gmd* and *gst1* were detected. Interestingly, Yang et al. (2020) reported *gmd* and *rmd* (oxidoreductase Rmd) mutations that occurred in *P. aeruginosa* phage‐resistant mutants when treated with a cocktail of five phages. However, they found that mutations in *wzy* (B‐band O‐antigen polymerase) and *migA* (Alpha‐ 1,6‐rhamnosyltransferase), both involved in LPS biosynthesis, were probably the essential genes for bacterial resistance to the five‐phage cocktail. Why so many other mutations occur for single phage infections is not clear, but seems to indicate that the cocktail of phages somehow imposes a more stringent selection against a diverse range of mutants surviving in the population. This is certainly a desirable outcome for any kind of therapy; however, completely unwanted is the ability of *Pss* to be able to rapidly break phage infection via just one, maximum two, mutations in the LPS genes *gst1* and *gmd*. Whether this occurs *in planta* is yet to be investigated, but given the small impacts on in vitro fitness (and no change for *gst1* mutation), this remains a distinct possibility. If the same outcome is borne out then that emphasizes the need to carefully design phage cocktails for either multiple adhesion targets (to minimize the opportunity for multi‐mutation sites to emerge) or using evolved or engineered phage genotypes that target changes in the same bacterial receptor. Indeed, when a phage evolved via interaction with *Pss* was tested along with the 5C cocktail, killing of the pathogen was observed without the emergence of phage‐resistant mutants.

This study has addressed biological questions relating to making an effective phage cocktail to treat a complex tree disease like cherry canker. It would appear that phages with different receptor dependency should be utilized where possible to maximize the efficacy of phages in treating a mixed pathogen population and to minimize the emergence of phage‐resistant mutants. However, these observations indicate that phages can be trained to target phage‐resistant mutants and this may be sufficient to prevent the emergence of bacterial resistance in vitro. This approach should help to inform disease resistance management strategies and the development of phage‐based crop protection measures in the future as well as create a platform for testing phages with other emerging antimicrobial technologies such as bacteriocins and synthetic communities.

## AUTHOR CONTRIBUTIONS

**Mojgan Rabiey:** Conceptualization; investigation; funding acquisition; writing – original draft; methodology; validation; visualization; writing – review and editing; project administration; supervision; data curation; formal analysis. **Emily R. Grace:** Investigation; writing – review and editing; methodology; formal analysis; visualization; validation. **Paulina Pawlos:** Methodology; writing – review and editing. **Muscab Bihi:** Writing – review and editing; methodology. **Haleem Ahmed:** Methodology; writing – review and editing. **Georgina E. Hampson:** Methodology; writing – review and editing. **Amna Al Riyami:** Methodology; writing – review and editing. **Leena Alharbi:** Methodology; writing – review and editing. **Rosa Sanchez‐Lucas:** Methodology; writing – review and editing. **Naina Korotania:** Methodology; writing – review and editing. **Maria Laura Ciusa:** Methodology; writing – review and editing; investigation; visualization. **Olivia Mosley:** Formal analysis; writing – review and editing. **Michelle T. Hulin:** Formal analysis; writing – review and editing. **Laura Baxter:** Formal analysis; writing – review and editing. **Sabrine Dhaouadi:** Formal analysis; writing – review and editing; methodology. **Diana Vinchira‐Villarraga:** Methodology; writing – review and editing; formal analysis. **Robert W. Jackson:** Conceptualization; investigation; funding acquisition; writing – original draft; writing – review and editing; visualization; validation; methodology; project administration; supervision; resources; formal analysis.

## CONFLICT OF INTEREST STATEMENT

The authors declare that they have no conflict of interest.

## Supporting information


Appendix S1.

Table S2.

## Data Availability

The datasets supporting the conclusions of this article are included within the article and its Supplementary Materials.
