# Supplementary material for: Coevolutionary analysis of Pseudomonas syringae–phage interactions to help with rational design of phage treatments
Source: Microb Biotechnol. 2024 Jun 12;17(6):e14489. doi: 10.1111/1751-7915.14489 (PMC11167607; doi:10.1111/1751-7915.14489)
Supplement: Supplementary file 1 — Appendix S1. [file MBT2-17-e14489-s002.docx]

**Supporting information**

**Coevolutionary analysis of *Pseudomonas syringae*-phage interactions to help with rational design of phage treatments**

Mojgan Rabiey^1,2Ψ*^, Emily R. Grace^2Ψ^, Paulina Pawlos^2^, Muscab Bihi^2^, Haleem Ahmed^2^, Georgina E. Hampson^2^, Amna Al Riyami^2^, Leena Alharbi^2^, Rosa Sanchez-Lucas^2^, Naina Korotania^2^, Maria Laura Ciusa^2^, Olivia Mosley^2^, Michelle T. Hulin^3^, Laura Baxter^4^, Sabrine Dhaouadi^2^, Diana Vinchira-Villarraga^2^ and Robert W. Jackson^2^

^1^School of Life Sciences, Gibbet Hill Campus, University of Warwick, Coventry , UK

^2^School of Biosciences and the Birmingham Institute of Forest Research, University of Birmingham, UK

^3^Department of Plant Soil & Microbial Sciences, Michigan State University, East Lansing, Michigan, US

^4^Bioinformatics Research Technology Platform, University of Warwick, Coventry, UK

^Ψ^These authors contributed equally to this work.

Corresponding author: *Mojgan Rabiey, [Mojgan.rabiey@warwick.ac.uk](mailto:Mojgan.rabiey@warwick.ac.uk)

**4 Supporting Tables**

**9 Supporting Figures**

**Table S1. List of primers used for construction of *Pseudomonas* *syringae* pv. *syringae* strain 9097 (accession number CP026568) marker less mutants in this study.**

| Gene | F/R | Primer sequence | Restriction enzyme | Size (bp) | Δ gene size (bp) | WT gene size (bp) |
| --- | --- | --- | --- | --- | --- | --- |
| Glycosyltransferase family 1 protein | F1 | GGTGGTTCTAGAtcaaggcccggccgcaggttgtg | XbaI | 480 | 979 | 1959 |
|  | R1 | ctgaataccatcattcgtttccgcagccgtgatctctgattcag | | | |  |
|  | F2 | ctgaatcagagatcacggctgcggaaacgaatgatggtattcag | | | |  |
|  | R2 | GGTGGTGGATCCaaacccctgccattcaggtttg | BamH1 | 500 |  |  |
| Lipopolysaccharide kinase | F1 | GGTGGTTCTAGAaagtccgagtggcagaacctcaag | Xbal | 488 | 993 | 1354 |
|  | R1 | tcggcaactcgagggtacggccgacgatatgtcgaatacgtttc | | | |  |
|  | F2 | gaaacgtattcgacatatcgtcggccgtaccctcgagttgccga | | 505 |  |  |
|  | R2 | GGTGGTAAGCTTgttcagcacgaatcccggcgcc | HindIII |  |  |  |
| Glucose-1-phosphate thymidylyltransferase | F1 | GGTGGTTCTAGAatgtaacacggctggcgttcac | Xbal | 500 | 988 | 1911 |
|  | R1 | ctggatgaaattcagcgctaagatccgggcgactttccacacat | | | |  |
|  | F2 | atgtgtggaaagtcgcccggatcttagcgctgaatttcatccag | | 488 |  |  |
|  | R2 | GGTGGTGGATCCtcaggccagtggtgcaaaggcg | BamHI |  |  |  |
| phosphomannomutase/phosphoglucomutase | F1 | GGTGGTTCTAGAatgttgagcagcccggtgttgac | Xbal | 502 | 1398 | 4073 |
|  | R1 | tcagcctcaggcagcgcctgtccgcgatgccgcctctaatgtag | | | |  |
|  | F2 | ctacattagaggcggcatcgcggacaggcgctgcctgaggctga | | 489 |  |  |
|  | R2 | GGTGGTAAGCTTcgctgccagtcagccggtggtgg | HindIII |  |  |  |
| autotransporter outer membrane beta-barrel domain-containing protein | F1 | GGTGGTTCTAGAcattcatgtggacgcatcccggc | Xbal | 498 | 2745 | 3754 |
|  | R1 | gttgtcgcggcgcgccactcaacggatcagcaccgcgaggggtgg | | | |  |
|  | F2 | ccacccctcgcggtgctgatccgttgagtggcgcgccgcgacaac | | 511 |  |  |
|  | R2 | GGTGGTGCGATCGCctgacgctctcgtcgatcggctac | SfaAI |  |  |  |
| ATP-grasp domain-containing protein | F1 | GGTGGTTCTAGAtgcgacgcgaccatcccggccg | Xbal | 479 | 4665 | 5898 |
|  | R1 | cggtgtccaggcgcaggtcatttatttgccgggcgcggaaatac | | | |  |
|  | F2 | gtatttccgcgcccggcaaataaatgacctgcgcctggacaccg | | | |  |
|  | R2 | GGTGGTAAGCTTcagggccacggccagcgacgag | HindIII | 474 |  |  |
| M13 | F | tgtaaaacgacggccagt |  | 400 |  |  |
|  | R | caggaaacagctatgacc |  |  |  |  |

**Table S2. Statistical analysis of all figures in the main part of the results.** To evaluate differences in the killing curves and growth curves of the bacterial isolates in the absence and presence of phages, individually or in cocktail 5C, ANOVA test was used at specific time points. Post hoc Tukey test were applied to evaluate differences among treatments (p<0.05). The statistical analysis for Figure 4 presented in the table corresponds to the comparison of differences between the proportion of phage-resistant bacteria in each coevolution generation. All the statistical analysis were carried out in GraphPad prism 9 (Boston, Massachusetts USA, [www.graphpad.com](http://www.graphpad.com)). The data are supplied in a separate Excel spreadsheet.

**Table S3. Mutations identified in *Pseudomonas* *syringae* pv. *syringae* strain 9097 (*Pss,* accession number CP026568)** colonies collected three times (T1, T2 and T3) during the course of 66 h killing curve assay, with MR1, MR4, MR6, MR14, MR15 and cocktail 5 (5C). Six colonies were whole genome sequenced at each time point (e.g. T1-1.1, -1.2, -2.1, -2.2, -3.1 and -3.2) and variant calling was employed. Location: the specific position in the genome where the variant is located, Reference: nucleotide found at a particular position in the *Pss* genome, Alternative: any nucleotide that differs from the reference at a particular position in the mutant.

| **Generation** | **Location** | **Strand** | **Reference** | **Alternative** | **Gene name** | **Product** |
| --- | --- | --- | --- | --- | --- | --- |
| **Pss-MR1 interaction** | | | | | | |
| MR1-T1-1.1 | 1061822 | - | GC | G | AVB24517.1 | GDP-mannose 4,6-dehydratase |
| MR1-T2-1.1 | 609421 | + | GA | G | AVB24142.1 | glycosyltransferase family 1 |
| MR1-T2-1.1 | 4765810 | - | T | C | AVB27436.1 | FtsW |
| MR1-T2-1.2 | 609421 | + | GA | G | AVB24142.1 | glycosyltransferase family 1 |
| MR1-T2-1.2 | 4765810 | - | T | C | AVB27436.1 | FtsW |
| MR1-T2-2.1 | 609421 | + | GA | G | AVB24142.1 | glycosyltransferase family 1 |
| MR1-T2-2.1 | 4765810 | - | T | C | AVB27436.1 | FtsW |
| MR1-T2-2.2 | 609421 | + | GA | G | AVB24142.1 | glycosyltransferase family 1 |
| MR1-T2-2.2 | 4765810 | - | T | C | AVB27436.1 | FtsW |
| MR1-T2-3.1 | 1061822 | - | GC | G | AVB24517.1 | GDP-mannose 4,6-dehydratase |
| MR1-T3-1.1 | 1294998 | + | C | T | AVB24723.1 | antibiotic acetyltransferase |
| MR1-T3-1.2 | 1061822 | - | GC | G | AVB24517.1 | GDP-mannose 4,6-dehydratase |
| MR1-T3-1.2 | 1294998 | + | C | T | AVB24723.1 | antibiotic acetyltransferase |
| MR1-T3-2.1 | 1061822 | - | GC | G | AVB24517.1 | GDP-mannose 4,6-dehydratase |
| MR1-T3-2.1 | 1294998 | + | C | T | AVB24723.1 | antibiotic acetyltransferase |
| MR1-T3-2.2 | 1061822 | - | GC | G | AVB24517.1 | GDP-mannose 4,6-dehydratase |
| MR1-T3-2.2 | 1294998 | + | C | T | AVB24723.1 | antibiotic acetyltransferase |
| MR1-T3-3.1 | 609421 | + | GA | G | AVB24142.1 | glycosyltransferase family 1 |
| MR1-T3-3.1 | 4606697 | + | GCCCTCTTCGCGAGCAAGCTCGCTCCCACAAGGCCCCGCAAACTCCCGTGGGAGCGAGCTTGCTCGCGAAGGCTATATGCCTGACGCAGAAAATCCATTGACTACACCT | G | AVB27313.1 | ATP-grasp domain-containing |
| MR1-T3-3.1 | 4765810 | - | T | C | AVB27436.1 | FtsW |
| MR1-T3-3.2 | 1061822 | - | GC | G | AVB24517.1 | GDP-mannose 4,6-dehydratase |
| MR1-T3-3.2 | 1294998 | + | C | T | AVB24723.1 | antibiotic acetyltransferase |
| **Pss-MR4 interaction** | | | | | | |
| MR4-T1-1.2 | 1061822 | - | GC | G | AVB24517.1 | GDP-mannose 4,6-dehydratase |
| MR4-T1-2.1 | 4606697 | + | GA | G | AVB24142.1 | glycosyltransferase family 1 |
| MR4-T1-2.2 | 609421 | + | GCCCTCTTCGCGAGCAAGCTCGCTCCCACAAGGCCCCGCAAACTCCCGTGGGAGCGAGCTTGCTCGCGAAGGCTATATGCCTGACGCAGAAAATCCATTGACTACACCT | G | AVB27313.1 | ATP-grasp domain-containing protein |
| MR4-T1-2.2 | 4765810 | - | T | C | AVB27436.1 | FtsW |
| MR4-T1-3.2 | 1061822 | - | GC | G | AVB24517.1 | GDP-mannose 4,6-dehydratase |
| MR4-T2-1.1 | 609421 | + | GA | G | AVB24142.1 | glycosyltransferase family 1 |
| MR4-T2-1.2 | 4765810 | - | T | C | AVB27436.1 | FtsW |
| MR4-T2-3.1 | 4606697 | + | GCCCTCTTCGCGAGCAAGCTCGCTCCCACAAGGCCCCGCAAACTCCCGTGGGAGCGAGCTTGCTCGCGAAGGCTATATGCCTGACGCAGAAAATCCATTGACTACACCT | G | AVB27313.1 | ATP-grasp domain-containing protein |
| MR4-T2-3.1 | 4765810 | - | T | C | AVB27436.1 | FtsW |
| MR4-T2-3.2 | 609421 | + | GA | G | AVB24142.1 | glycosyltransferase family 1 |
| MR4-T2-3.2 | 4765810 | - | T | C | AVB27436.1 | FtsW |
| MR4-T3-1.1 | 609421 | + | GA | G | AVB24142.1 | glycosyltransferase family 1 |
| MR4-T3-1.1 | 4606697 | + | GCCCTCTTCGCGAGCAAGCTCGCTCCCACAAGGCCCCGCAAACTCCCGTGGGAGCGAGCTTGCTCGCGAAGGCTATATGCCTGACGCAGAAAATCCATTGACTACACCT | G | AVB27313.1 | ATP-grasp domain-containing protein |
| MR4-T3-1.1 | 4765810 | - | T | C | AVB27436.1 | FtsW |
| MR4-T3-1.2 | 609421 | + | GA | G | AVB24142.1 | glycosyltransferase family 1 |
| MR4-T3-1.2 | 4765810 | - | T | C | AVB27436.1 | FtsW |
| MR4-T3-2.1 | 609421 | + | GA | G | AVB24142.1 | glycosyltransferase family 1 |
| MR4-T3-2.1 | 4765810 | - | T | C | AVB27436.1 | FtsW |
| MR4-T3-2.2 | 609421 | + | GA | G | AVB24142.1 | glycosyltransferase family 1 |
| MR4-T3-2.2 | 4765810 | - | T | C | AVB27436.1 | FtsW |
| MR4-T3-3.1 | 609421 | + | GA | G | AVB24142.1 | glycosyltransferase family 1 |
| MR4-T3-3.1 | 4606697 | + | GCCCTCTTCGCGAGCAAGCTCGCTCCCACAAGGCCCCGCAAACTCCCGTGGGAGCGAGCTTGCTCGCGAAGGCTATATGCCTGACGCAGAAAATCCATTGACTACACCT | G | AVB27313.1 | ATP-grasp domain-containing protein |
| MR4-T3-3.1 | 4765810 | - | T | C | AVB27436.1 | FtsW |
| MR4-T3-3.2 | 609421 | + | GA | G | AVB24142.1 | glycosyltransferase family 1 |
| MR4-T3-3.2 | 4765810 | - | T | C | AVB27436.1 | FtsW |
| **Pss-MR6 interaction** | | | | | | |
| MR6-T1-1.1 | 1061822 | - | GC | G | AVB24517.1 | GDP-mannose 4,6-dehydratase |
| MR6-T1-1.2 | 5619948 | + | T | C | AVB28161.1 | hypothetical protein |
| MR6-T1-2.1 | 1061822 | - | GC | G | AVB24517.1 | GDP-mannose 4,6-dehydratase; |
| MR6-T1-3.1 | 609421 | + | GA | G | AVB24142.1 | glycosyltransferase family 1 |
| MR6-T1-3.1 | 4765810 | - | T | C | AVB27436.1 | FtsW |
| MR6-T1-3.2 | 609421 | + | GA | G | AVB24142.1 | glycosyltransferase family 1 |
| MR6-T1-3.2 | 4765810 | - | T | C | AVB27436.1 | FtsW |
| MR6-T2-1.1 | 609421 | + | GA | G | AVB24142.1 | glycosyltransferase family 1 |
| MR6-T2-1.1 | 4765810 | - | T | C | AVB27436.1 | FtsW |
| MR6-T2-1.2 | 609421 | + | GA | G | AVB24142.1 | glycosyltransferase family 1 |
| MR6-T2-1.2 | 4765810 | - | T | C | AVB27436.1 | FtsW |
| MR6-T2-3.1 | 609421 | + | GA | G | AVB24142.1 | glycosyltransferase family 1 |
| MR6-T2-3.1 | 4765810 | - | T | C | AVB27436.1 | FtsW |
| MR6-T2-3.2 | 609421 | + | GA | G | AVB24142.1 | glycosyltransferase family 1 |
| MR6-T2-3.2 | 4765810 | - | T | C | AVB27436.1 | FtsW |
| MR6-T3-1.1 | 609421 | + | GA | G | AVB24142.1 | glycosyltransferase family 1 |
| MR6-T3-1.1 | 4606716 | + | T | C | AVB27313.1 | ATP-grasp domain-containing protein |
| MR6-T3-1.1 | 4765810 | - | T | C | AVB27436.1 | FtsW |
| MR6-T3-1.2 | 609421 | + | GA | G | AVB24142.1 | glycosyltransferase family 1 |
| MR6-T3-1.2 | 4765810 | - | T | C | AVB27436.1 | FtsW |
| MR6-T3-2.1 | 609421 | + | GA | G | AVB24142.1 | glycosyltransferase family 1 |
| MR6-T3-2.1 | 4765810 | - | T | C | AVB27436.1 | FtsW |
| MR6-T3-2.2 | 609421 | + | GA | G | AVB24142.1 | glycosyltransferase family 1 |
| MR6-T3-2.2 | 4606716 | + | T | C | AVB27313.1 | ATP-grasp domain-containing protein |
| MR6-T3-2.2 | 4765810 | - | T | C | AVB27436.1 | FtsW |
| MR6-T3-3.1 | 609421 | + | GA | G | AVB24142.1 | glycosyltransferase family 1 |
| MR6-T3-3.1 | 4765810 | - | T | C | AVB27436.1 | FtsW |
| MR6-T3-3.2 | 609421 | + | GA | G | AVB24142.1 | glycosyltransferase family 1 |
| MR6-T3-3.2 | 4765810 | - | T | C | AVB27436.1 | FtsW |
| **Pss-MR14 interaction** | | | | | | |
| MR14-T1-1.1 | 1061822 | - | GC | G | AVB24517.1 | GDP-mannose 4,6-dehydratase; |
| MR14-T1-2.2 | 609421 | + | GA | G | AVB24142.1 | glycosyltransferase family 1 |
| MR14-T1-2.2 | 4765810 | - | T | C | AVB27436.1 | FtsW |
| MR14-T1-3.1 | 609421 | + | GA | G | AVB24142.1 | glycosyltransferase family 1 |
| MR14-T1-3.1 | 4765810 | - | T | C | AVB27436.1 | FtsW |
| MR14-T1-3.2 | 609421 | + | GA | G | AVB24142.1 | glycosyltransferase family 1 |
| MR14-T1-3.2 | 4765810 | - | T | C | AVB27436.1 | FtsW |
| MR14-T2-1.1 | 609421 | + | GA | G | AVB24142.1 | glycosyltransferase family 1 |
| MR14-T2-1.1 | 4606716 | + | T | C | AVB27313.1 | ATP-grasp domain-containing protein |
| MR14-T2-1.1 | 4765810 | - | T | C | AVB27436.1 | FtsW |
| MR14-T2-1.2 | 609421 | + | GA | G | AVB24142.1 | glycosyltransferase family 1 |
| MR14-T2-1.2 | 4765810 | - | T | C | AVB27436.1 | FtsW |
| MR14-T2-2.1 | 609421 | + | GA | G | AVB24142.1 | glycosyltransferase family 1 |
| MR14-T2-2.1 | 4765810 | - | T | C | AVB27436.1 | FtsW |
| MR14-T2-2.2 | 609421 | + | GA | G | AVB24142.1 | glycosyltransferase family 1 |
| MR14-T2-2.2 | 4765810 | - | T | C | AVB27436.1 | FtsW |
| MR14-T2-3.1 | 609421 | + | GA | G | AVB24142.1 | glycosyltransferase family 1 |
| MR14-T2-3.1 | 4765810 | - | T | C | AVB27436.1 | FtsW |
| MR14-T2-3.2 | 1065543 | + | C | G | AVB24520.1 | FtsW |
| MR14-T2-3.2 | 1294282 | + | A | G | AVB24722.1 | glycosyltransferase family 2 |
| MR14-T3-1.1 | 609421 | + | GA | G | AVB24142.1 | glycosyltransferase family 1 |
| MR14-T3-1.1 | 4765810 | - | T | C | AVB27436.1 | FtsW |
| MR14-T3-1.2 | 609421 | + | GA | G | AVB24142.1 | glycosyltransferase family 1 |
| MR14-T3-1.2 | 4765810 | - | T | C | AVB27436.1 | FtsW |
| MR14-T3-2.1 | 609421 | + | GA | G | AVB24142.1 | glycosyltransferase family 1 |
| MR14-T3-2.1 | 4606697 | + | GCCCTCTTCGCGAGCAAGCTCGCTCCCACAAGGCCCCGCAAACTCCCGTGGGAGCGAGCTTGCTCGCGAAGGCTATATGCCTGACGCAGAAAATCCATTGACTACACCT | G | AVB27313.1 | ATP-grasp domain-containing protein |
| MR14-T3-2.1 | 4765810 | - | T | C | AVB27436.1 | FtsW |
| MR14-T3-2.2 | 609421 | + | GA | G | AVB24142.1 | glycosyltransferase family 1 |
| MR14-T3-2.2 | 4606716 | + | T | C | AVB27313.1 | ATP-grasp domain-containing protein |
| MR14-T3-2.2 | 4765810 | - | T | C | AVB27436.1 | FtsW |
| MR14-T3-3.1 | 609421 | + | GA | G | AVB24142.1 | glycosyltransferase family 1 |
| MR14-T3-3.1 | 4765810 | - | T | C | AVB27436.1 | FtsW |
| MR14-T3-3.2 | 609421 | + | GA | G | AVB24142.1 | glycosyltransferase family 1 |
| MR14-T3-3.2 | 4765810 | - | T | C | AVB27436.1 | FtsW |
| **Pss-MR15 interaction** | | | | | | |
| MR15-T1-1.1 | 1061822 | GC | G | 1366.01 | AVB24517.1 | GDP-mannose 4,6-dehydratase; |
| MR15-T1-1.1 | 4606697 | GCCCTCTTCGCGAGCAAGCTCGCTCCCACAAGGCCCCGCAAACTCCCGTGGGAGCGAGCTTGCTCGCGAAGGCTATATGCCTGACGCAGAAAATCCATTGACTACACCT | G | 125.01 | AVB27313.1 | ATP-grasp domain-containing protein |
| MR15-T1-1.2 | 1065543 | C | G | 1604.04 | AVB24520.1 | FtsW |
| MR15-T1-2.1 | 1061822 | GC | G | 2131.01 | AVB24517.1 | GDP-mannose 4,6-dehydratase; |
| MR15-T1-3.1 | 609421 | GA | G | 5249.01 | AVB24142.1 | glycosyltransferase family 1 |
| MR15-T1-3.1 | 4606697 | GCCCTCTTCGCGAGCAAGCTCGCTCCCACAAGGCCCCGCAAACTCCCGTGGGAGCGAGCTTGCTCGCGAAGGCTATATGCCTGACGCAGAAAATCCATTGACTACACCT | G | 367.01 | AVB27313.1 | ATP-grasp domain-containing protein |
| MR15-T1-3.1 | 4765810 | T | C | 2452.04 | AVB27436.1 | FtsW |
| MR15-T2-1.1 | 609421 | GA | G | 4977.01 | AVB24142.1 | glycosyltransferase family 1 |
| MR15-T2-1.1 | 4765810 | T | C | 3211.04 | AVB27436.1 | FtsW |
| MR15-T2-1.2 | 609421 | GA | G | 3546.01 | AVB24142.1 | glycosyltransferase family 1 |
| MR15-T2-1.2 | 4765810 | T | C | 2651.04 | AVB27436.1 | FtsW |
| MR15-T2-2.1 | 609421 | GA | G | 3342.01 | AVB24142.1 | glycosyltransferase family 1 |
| MR15-T2-2.1 | 4765810 | T | C | 1924.04 | AVB27436.1 | FtsW |
| MR15-T2-2.2 | 609421 | GA | G | 4562.01 | AVB24142.1 | glycosyltransferase family 1 |
| MR15-T2-2.2 | 4765810 | T | C | 3427.04 | AVB27436.1 | FtsW |
| MR15-T2-3.1 | 609421 | GA | G | 4239.01 | AVB24142.1 | glycosyltransferase family 1 |
| MR15-T2-3.1 | 4765810 | T | C | 2180.04 | AVB27436.1 | FtsW |
| MR15-T2-3.2 | 609421 | GA | G | 2948.01 | AVB24142.1 | glycosyltransferase family 1 |
| MR15-T2-3.2 | 4765810 | T | C | 2372.04 | AVB27436.1 | FtsW |
| MR15-T3-1.1 | 609376 | G | GA | 3361.01 | AVB24142.1 | glycosyltransferase family 1 |
| MR15-T3-1.1 | 1061822 | GC | G | 2522.01 | AVB24517.1 | GDP-mannose 4,6-dehydratase; |
| MR15-T3-1.2 | 609421 | GA | G | 2981.01 | AVB24142.1 | glycosyltransferase family 1 |
| MR15-T3-1.2 | 4765810 | T | C | 2159.04 | AVB27436.1 | FtsW |
| MR15-T3-2.1 | 609421 | GA | G | 3952.01 | AVB24142.1 | glycosyltransferase family 1 |
| MR15-T3-2.1 | 4765810 | T | C | 1747.04 | AVB27436.1 | FtsW |
| MR15-T3-2.2 | 609421 | GA | G | 3268.01 | AVB24142.1 | glycosyltransferase family 1 |
| MR15-T3-2.2 | 4765810 | T | C | 1547.04 | AVB27436.1 | FtsW |
| MR15-T3-3.1 | 609421 | GA | G | 4742.01 | AVB24142.1 | glycosyltransferase family 1 |
| MR15-T3-3.1 | 4606716 | T | C | 152.04 | AVB27313.1 | ATP-grasp domain-containing protein |
| MR15-T3-3.1 | 4765810 | T | C | 2072.04 | AVB27313.1 | FtsW |
| MR15-T3-3.2 | 609421 | GA | G | 5212.01 | AVB24142.1 | glycosyltransferase family 1 |
| MR15-T3-3.2 | 4765810 | T | C | 2860.04 | AVB27436.1 | FtsW |
| **Pss-Cocktail 5 interaction** | | | | | | |
| 5C-T1-1.1 | 1065543 | + | C | G | AVB24520.1 | hypothetical protein |
| 5C-T1-1.2 | 1061822 | - | GC | G | AVB24517.1 | GDP-mannose 4,6-dehydratase; |
| 5C-T2-1.2 | 609376 | + | G | GA | AVB24142.1 | glycosyltransferase family 1 |
| 5C-T2-1.2 | 1061822 | - | GC | G | AVB24517.1 | GDP-mannose 4,6-dehydratase; |
| 5C-T2-2.1 | 609376 | + | G | GA | AVB24142.1 | glycosyltransferase family 1 |
| 5C-T2-2.1 | 1061822 | - | GC | G | AVB24517.1 | GDP-mannose 4,6-dehydratase; |
| 5C-T2-2.2 | 609376 | + | G | GA | AVB24142.1 | glycosyltransferase family 1 |
| 5C-T2-2.2 | 1061822 | - | GC | G | AVB24517.1 | GDP-mannose 4,6-dehydratase; |
| 5C-T2-3.1 | 609421 | + | GA | G | AVB24142.1 | glycosyltransferase family 1 |
| 5C-T2-3.1 | 4765810 | - | T | C | AVB27436.1 | FtsW |
| 5C-T3-2.1 | 609421 | + | GA | G | AVB24142.1 | glycosyltransferase family 1 |
| 5C-T3-2.1 | 4765810 | - | T | C | AVB27436.1 | FtsW |

**Table S4. Mutations identified in *Pseudomonas* *syringae* pv. *syringae* strain 9097 (*Pss*)** colonies collected, at generation 2B, 4B, 6B, 8B and 10B, during the experimental coevolution of *Pss* with phage MR1, MR4, MR6, MR14, MR15 and cocktail 5 (5C). Three colonies were whole genome sequenced at each generation and variant calling was employed. Location: the specific position in the genome where the variant is located, Reference: nucleotide found at a particular position in the *Pss* genome, Alternative: any nucleotide that differs from the reference at a particular position in the mutant.

| **Generation** | **Location** | **Strand** | **Reference** | **Alternative** | **Gene name** | **Product** |
| --- | --- | --- | --- | --- | --- | --- |
| ***Pss*-coevolved with phage MR1** | | | | | | |
| MR1-1-2B | 595225 | + | TGGCGAGCGCAAGGTCGAC | T | AVB24135.1 | lipopolysaccharide kinase |
| MR1-1-4B | 609736 | + | C | T | AVB24142.1 | glycosyltransferase family 1 |
| MR1-2-2B | 595225 | + | TGGCGAGCGCAAGGTCGAC | T | AVB24135.1 | lipopolysaccharide kinase |
| MR1-2-4B | 3936786 | + | G | A | AVB26749.1 | hypothetical protein |
| MR1-2-4B | 4606716 | + | T | C | AVB27313.1 | ATP-grasp domain-containing |
| MR1-2-6B | 595225 | + | TGGCGAGCGCAAGGTCGAC | T | AVB24135.1 | lipopolysaccharide kinase |
| MR1-2-6B | 609736 | + | C | T | AVB24142.1 | glycosyltransferase family 1 |
| MR1-2-6B | 3936786 | + | G | A | AVB24135.1 | hypothetical protein |
| MR1-3-4B | 4606717 | + | C | A | AVB27313.1 | ATP-grasp domain-containing protein |
| MR1-3-6B | 3963331 | - | A | C | AVB26776.1 | RNA polymerase sigma factor FliA |
| MR1-3-6B | 4606721 | + | C | T | AVB27313.1 | ATP-grasp domain-containing protein |
| MR1-1-8B | 2417 | + | AC | A | AVB23630.1 | DNA replication and repair protein RecF |
| MR1-1-8B | 3565 | + | G | A | AVB23631.1 | gyrB |
| MR1-2-8B | 2421 | + | C | T | AVB23630.1 | DNA replication and repair protein RecF |
| MR1-2-8B | 3577 | + | T | A | AVB23631.1 | gyrB |
| MR1-3-8B | 2424 | + | A | C | AVB23630.1 | DNA replication and repair protein RecF |
| MR1-3-8B | 4606733 | + | C | G | AVB27313.1 | ATP-grasp domain-containing protein |
| ***Pss*-coevolved with phage MR4** | | | | | | |
| MR4-1-2B | 237896 | - | C | T | AVB28423.1 | phosphomannomutase/phosphoglucomutase |
| MR4-1-2B | 1068418 | - | GAGAAGGCCTTTCAAATAT | G | AVB24523.1 | glucose-1-phosphate thymidylyltransferase |
| MR4-1-2B | 1196150 | - | CT | C | AVB24642.1 | alginate export family protein |
| MR4-1-2B | 2959807 | + | GA | G | AVB26047.1 | D-alanine--poly(phosphoribitol) ligase subunit DltA |
| MR4-1-2B | 4202422 | - | TG | T | AVB26987.1 | DNA gyrase subunit A |
| MR4-1-4B | 237896 | - | C | T | AVB28423.1 | phosphomannomutase/phosphoglucomutase |
| MR4-1-4B | 1068418 | - | GAGAAGGCCTTTCAAATAT | G | AVB24523.1 | glucose-1-phosphate thymidylyltransferase |
| MR4-1-4B | 2845964 | - | T | TGCCCTGCCCTGACTGCCAGTACTGACGAAACAGCTCCGGACGCCCGACGCGGTCGGCATACTCCCGAAATGCCGCCATGTA | AVB25964.1 | autotransporter outer membrane beta-barrel domain-containing protein |
| MR4-3-4B | 1388044 | . | C | G | AVB24802.1 | tRNA guanosine(34) transglycosylase Tgt |
| MR4-1-6B | 237896 | - | C | T | AVB28423.1 | phosphomannomutase/phosphoglucomutase |
| MR4-1-6B | 1068418 | - | GAGAAGGCCTTTCAAATAT | G | AVB24523.1 | glucose-1-phosphate thymidylyltransferase |
| MR4-2-6B | 594926 | . | G | A | AVB24135.1 | lipopolysaccharide kinase |
| MR4-2-6B | 608911 | . | C | T | AVB24142.1 | glycosyltransferase family 1 protein |
| MR4-2-6B | 4584979 | . | G | A | AVB27294.1 | RNA polymerase subunit sigma |
| MR4-3-6B | 608911 | . | C | T | AVB24142.1 | glycosyltransferase family 1 protein |
| MR4-3-6B | 4726228 | . | G | A | AVB27401.1 | InaA protein |
| MR4-1-8B | 237896 | - | C | T | AVB28423.1 | phosphomannomutase/phosphoglucomutase |
| MR4-1-8B | 1068418 | - | GAGAAGGCCTTTCAAATAT | G | AVB24523.1 | glucose-1-phosphate thymidylyltransferase |
| MR4-1-8B | 2845964 | - | T | TGCCCTGCCCTGACTGCCAGTACTGACGAAACAGCTCCGGACGCCCGACGCGGTCGGCATACTCCCGAAATGCCGCCATGTA | AVB25964.1 | autotransporter outer membrane beta-barrel domain-containing protein |
| ***Pss*-coevolved with phage MR6** | | | | | | |
| MR6-1-2B | 609160 | + | C | T | AVB24142.1 | glycosyltransferase family 1 |
| MR6-1-2B | 1061908 | - | A | T | AVB24517.1 | GDP-mannose 4,6-dehydratase |
| MR6-2-2B | 1061534 | - | GGAGCTGGTACGGCGATA | G | AVB24517.1 | GDP-mannose 4,6-dehydratase |
| MR6-1-4B | 609160 | + | C | T | AVB24142.1 | glycosyltransferase family 1 |
| MR6-1-4B | 1061908 | - | A | T | AVB24517.1 | GDP-mannose 4,6-dehydratase |
| MR6-2-4B | 608911 | + | C | T | AVB24142.1 | glycosyltransferase family 1 protein |
| MR6-2-4B | 4603704 | + | T | C | AVB27313.1 | ATP-grasp domain-containing protein |
| MR6-3-4B | 608911 | + | C | T | AVB24142.1 | glycosyltransferase family 1 |
| MR6-3-4B | 4603704 | + | T | C | AVB27313.1 | ATP-grasp domain-containing protein |
| MR6-3-4B | 4726228 | - | G | A | AVB27401.1 | InaA protein |
| MR6-1-6B | 609160 | + | C | T | AVB24142.1 | glycosyltransferase family 1 |
| MR6-1-6B | 1062089 | - | C | CCAGT | AVB24517.1 | GDP-mannose 4,6-dehydratase |
| MR6-1-6B | 4726610 | - | G | A | AVB27401.1 | InaA protein |
| MR6-1-8B | 609160 | + | C | T | AVB24142.1 | glycosyltransferase family 1 |
| MR6-1-8B | 1062089 | - | C | CCAGT | AVB24517.1 | GDP-mannose 4,6-dehydratase |
| MR6-1-8B | 4726610 | - | G | A | AVB27401.1 | InaA protein |
| MR6-1-10B | 609160 | + | C | T | AVB24142.1 | glycosyltransferase family 1 |
| MR6-1-10B | 620580 | + | G | T | AVB24152.1 | 3-deoxy-D-manno-octulosonic acid transferase |
| MR6-1-10B | 1062089 | - | C | CCAGT | AVB24517.1 | GDP-mannose 4,6-dehydratase |
| MR6-1-10B | 4726610 | - | G | A | AVB27401.1 | InaA protein |
| MR6-3-10B | 4603704 | + | C | G | AVB27313.1 | ATP-grasp domain-containing protein |
| ***Pss*-coevolved with phage MR14** | | | | | | |
| MR14-3-2B | 4603704 | + | T | C | AVB27313.1 | ATP-grasp domain-containing protein |
| MR14-1-4B | 595479 | + | A | G | AVB24135.1 | lipopolysaccharide kinase |
| MR14-1-4B | 609160 | + | C | T | AVB24142.1 | glycosyltransferase family 1 protein |
| MR14-1-4B | 1979700 | + | GTC | G | AVB25253.1 | mechanosensitive ion channel family protein |
| MR14-3-4B | 1661940 | - | GAGCGTCACGAACTGCATTCCCACGCCGGAGCGTGGGAACGATAGTCAACTGGCATCAACGGACGGACGTATTCTCTTCCTCCGGCATCTTCCTGAAATACGTCGAC | G |  | hypothetical protein |
| MR14-3-4B | 3425184 | - | T | C | AVB26331.1 | UTP--glucose-1-phosphate |
| MR14-3-4B | 4603704 | + | GCCCTCTTCGCGAGCAAGCTCGCTCCCACAAGGCCCCGCAAACTCCCGTGGGAGCGAGCTTGCTCGCGAAGGCTATATGCCTGACGCAGAAAATCCATTGACTACACCT | G | AVB27313.1 | ATP-grasp domain-containing protein |
| MR14-1-6B | 421616 | - | G | GA | AVB23992.1 | tatB |
| MR14-1-6B | 595479 | + | A | G | AVB24135.1 | lipopolysaccharide kinase |
| MR14-1-6B | 609160 | + | C | T | AVB24142.1 | glycosyltransferase family 1 |
| MR14-1-6B | 2294029 | + | A | ACT | AVB25508.1 | hypothetical protein |
| MR14-1-6B | 3398038 | + | C | CA | AVB28548.1 | ABC transporter substrate-binding protein |
| MR14-1-6B | 3671057 | + | T | TG | AVB26521.1 | leucyl/phenylalanyl-tRNA--protein transferase |
| MR14-3-6B | 594926 | + | A | G | AVB24135.1 | lipopolysaccharide kinase |
| MR14-3-6B | 608911 | + | C | T | AVB24142.1 | glycosyltransferase family 1 |
| MR14-3-6B | 4584979 | - | A | G | AVB27294.1 | RNA polymerase subunit sigma |
| MR14-1-8B | 595479 | + | A | G | AVB24135.1 | lipopolysaccharide kinase |
| MR14-1-8B | 609160 | + | C | T | AVB24142.1 | glycosyltransferase family 1 |
| MR14-2-8B | 594926 | + | A | G | AVB24135.1 | lipopolysaccharide kinase |
| MR14-2-8B | 608911 | + | C | T | AVB24142.1 | glycosyltransferase family 1 |
| MR14-2-8B | 4584979 | - | A | G | AVB27294.1 | RNA polymerase subunit sigma |
| MR14-3-8B | 594926 | + | G | A | AVB24135.1 | lipopolysaccharide kinase |
| MR14-3-8B | 608911 | + | C | T | AVB24142.1 | glycosyltransferase family 1 |
| MR14-3-8B | 1491089 | + | A | C | AVB24883.1 | lysine--tRNA ligase |
| MR14-3-8B | 3823542 | + | CGCT | C | AVB26651.1 | hypothetical protein |
| MR14-3-8B | 4726228 | - | G | A | AVB27401.1 | InaA protein |
| MR14-2-10B | 608911 | + | C | T | AVB24142.1 | glycosyltransferase family 1 |
| MR14-2-10B | 4726228 | - | G | A | AVB27401.1 | InaA protein |
| ***Pss*-coevolved with phage MR15** | | | | | | |
| MR15-1-2B | 2985588 | + | GCCAAA | G | AVB26062.1 | iron ABC transporter permease |
| MR15-1-2B | 4767733 | - | CACA | C | AVB27437.1 | UDP-N-acetylmuramoyl-L-alanine--D-glutamate ligase |
| MR15-3-2B | 3425184 | - | AGC | A | AVB26331.1 | UTP--glucose-1-phosphate |
| MR15-3-2B | 4603704 | + | C | G | AVB27313.1 | ATP-grasp domain-containing protein |
| MR15-1-4B | 609743 | + | T | A | AVB24142.1 | glycosyltransferase family 1 protein |
| MR15-1-4B | 2600300 | + | GTC | G | AVB25769.1 | ABC transporter ATP-binding protein |
| MR15-1-4B | 3714630 | + | T | C | AVB26557.1 | hypothetical protein |
| MR15-1-4B | 4189943 | - | GT | G | AVB26976.1 | polysaccharide biosynthesis protein |
| MR15-2-4B | 608911 | + | C | T | AVB24142.1 | glycosyltransferase family 1 |
| MR15-3-4B | 608911 | + | C | T | AVB24142.1 | glycosyltransferase family 1 |
| MR15-3-4B | 4188844 | - | G | A | AVB26976.1 | polysaccharide biosynthesis protein |
| MR15-3-4B | 4603704 | + | T | C | AVB27313.1 | ATP-grasp domain-containing protein |
| MR15-1-6B | 609743 | + | T | A | AVB24142.1 | glycosyltransferase family 1 |
| MR15-1-6B | 3714630 | + | T | C | AVB26557.1 | hypothetical protein |
| MR15-1-6B | 4189943 | - | GT | G | AVB26976.1 | polysaccharide biosynthesis protein |
| MR15-1-6B | 4757263 | + | GCA | G | AVB27428.1 | DUF721 domain-containing protein |
| MR15-2-6B | 594926 | + | G | A | AVB24135.1 | lipopolysaccharide kinase |
| MR15-2-6B | 608911 | + | C | T | AVB24142.1 | glycosyltransferase family 1 |
| MR15-2-6B | 3812923 | + | G | A | AVB26643.1 | undecaprenyl-phosphate glucose phosphotransferase |
| MR15-3-6B | 594926 | + | AC | A | AVB24135.1 | lipopolysaccharide kinase |
| MR15-3-6B | 608911 | + | C | T | AVB24142.1 | glycosyltransferase family 1 |
| MR15-3-6B | 3816746 | + | GTGATCGCGCGCT | G | AVB26646.1 | lipopolysaccharide biosynthesis protein |
| MR15-3-6B | 4188844 | - | G | A | AVB26976.1 | lipopolysaccharide biosynthesis protein |
| MR15-2-8B | 594926 | + | G | A | AVB24135.1 | lipopolysaccharide kinase |
| MR15-2-8B | 608911 | + | C | T | AVB24142.1 | glycosyltransferase family 1 |
| MR15-2-8B | 3816746 | + | G | GTGCTGGTGGTTCGCGCCCAGGACACCACGGTGCCGA | AVB26646.1 | lipopolysaccharide kinase |
| MR15-3-8B | 594926 | + | AC | A | AVB24135.1 | lipopolysaccharide kinase |
| MR15-3-8B | 608911 | + | C | T | AVB24142.1 | glycosyltransferase family 1 |
| MR15-3-8B | 1294440 | + | T | G | AVB24723.1 | antibiotic acetyltransferase |
| MR15-3-8B | 4188844 | - | G | A | AVB26976.1 | lipopolysaccharide biosynthesis protein |
| ***Pss*-coevolved with Cocktail 5** | | | | | | |
| MR5C-1-2B | 1061822 | - | GC | G | AVB24517.1 | GDP-mannose 4,6-dehydratase; |
| MR5C-1-4B | 609376 | + | G | GA | AVB24142.1 | glycosyltransferase family 1 |
| MR5C-1-6B | 1061822 | - | GC | G | AVB24517.1 | GDP-mannose 4,6-dehydratase; |
| MR5C-1-6B | 609376 | + | G | GA | AVB24142.1 | glycosyltransferase family 1 |
| MR5C-1-8B | 1061822 | - | GC | G | AVB24517.1 | GDP-mannose 4,6-dehydratase; |
| MR5C-1-8B | 609376 | + | G | GA | AVB24142.1 | glycosyltransferase family 1 |
| MR5C-3-8B | 1061822 | - | GC | G | AVB24517.1 | GDP-mannose 4,6-dehydratase; |
| MR5C-1-10B | 609421 | + | GA | G | AVB24142.1 | glycosyltransferase family 1 |
| MR5C-2-10B | 609421 | + | GA | G | AVB24142.1 | glycosyltransferase family 1 |


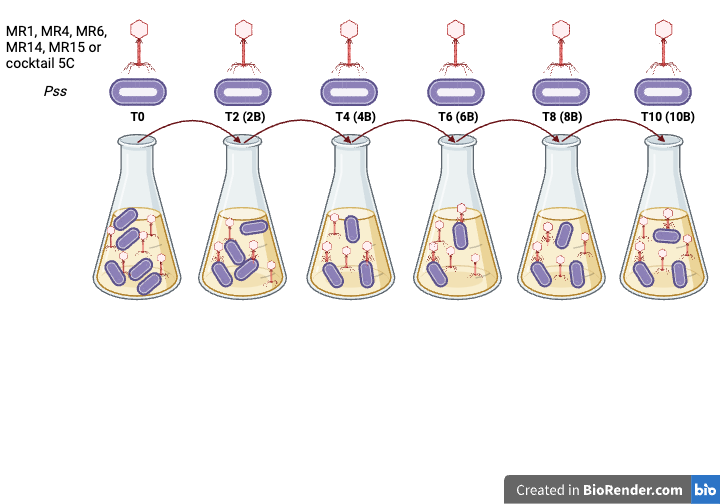


**Figure S1. The scheme of MR phage-*Pseudomonas syringae* pv. *syringae* (*Pss*) experimental coevolution in this study**. 5C: mixture (cocktail) of all 5 MR phages (created in BioRender.come).


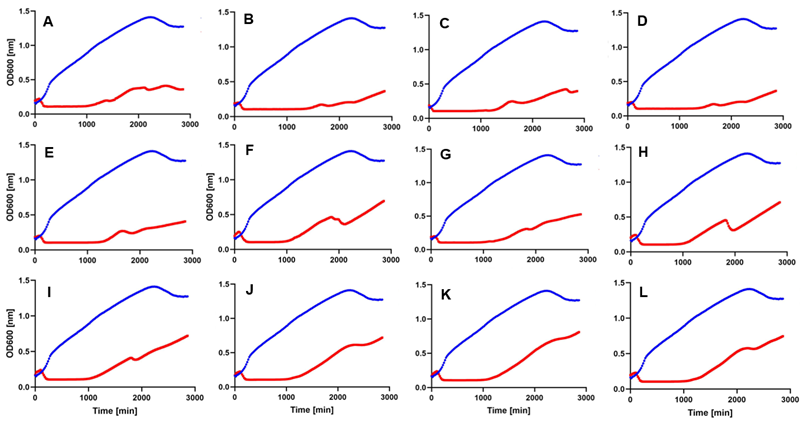


**Figure S2. Examples of *in vitro* killing curves of phage MR1, MR4, MR6, MR14 and MR15 individually or in combinations of, two, three and four, at multiplicity of infection of 0.01 on *Pseudomonas* *syringae* pv. *syringae* strain 9097 (*Pss*).** Top blue line is *Pss* and bottom red line is *Pss* and phage(s). **A.** MR1, **B.** MR4, **C.** MR14, **D.** MR15, **E.** MR1+MR4, **F.** MR1+MR14, **G.** MR4+MR14, **H.** MR14+MR15, **I.** MR1+MR4+MR14, **J.** MR1+MR4+MR15, **K.** MR4+MR14+MR15, **L.** MR1+MR4+MR14+MR15. Each experiment was repeated twice and each line represented three replicates.

**Figure S3. Panel I, *In vitro* killing curve of phage MR1, MR4, MR6, MR14, MR15 and cocktail 5C at multiplicity of infection of 0.01 on wildtype (WT) *Pseudomonas* *syringae* pv. *syringae* strain 9097 (*Pss*) during 66 h (3960 min). Panel II, *In vitro* killing curve of phage MR1, MR4, MR14, and MR15 on *Pss* WT** and *Pss* phage-resistant isolates collected at three time points (T1, T2, T3), during the 66 h killing curve assay with *Pss* and phage MR1 **(A-C)**, MR4 **(D-F)**, MR14 **(G-I)** and MR15 **(J-L). The killing curve of *Pss* phage resistant colonies for MR6, cocktail 5C and *Pss* control are shown in Figure 2.** The experiment was repeated twice and each line represented the mean of two replicates.

**Figure S4. Growth curve of** ***Pseudomonas* *syringae* pv. *syringae* strain 9097 (*Pss*) phage-resistant isolates collected at three time points (T1, T2, T3) during a 66 h killing curve assay with phage MR1 (A-C), MR4 (D-F), MR14 (G-I), MR15 (J-L).** The experiment was repeated twice and each line represented the mean of two replicates.


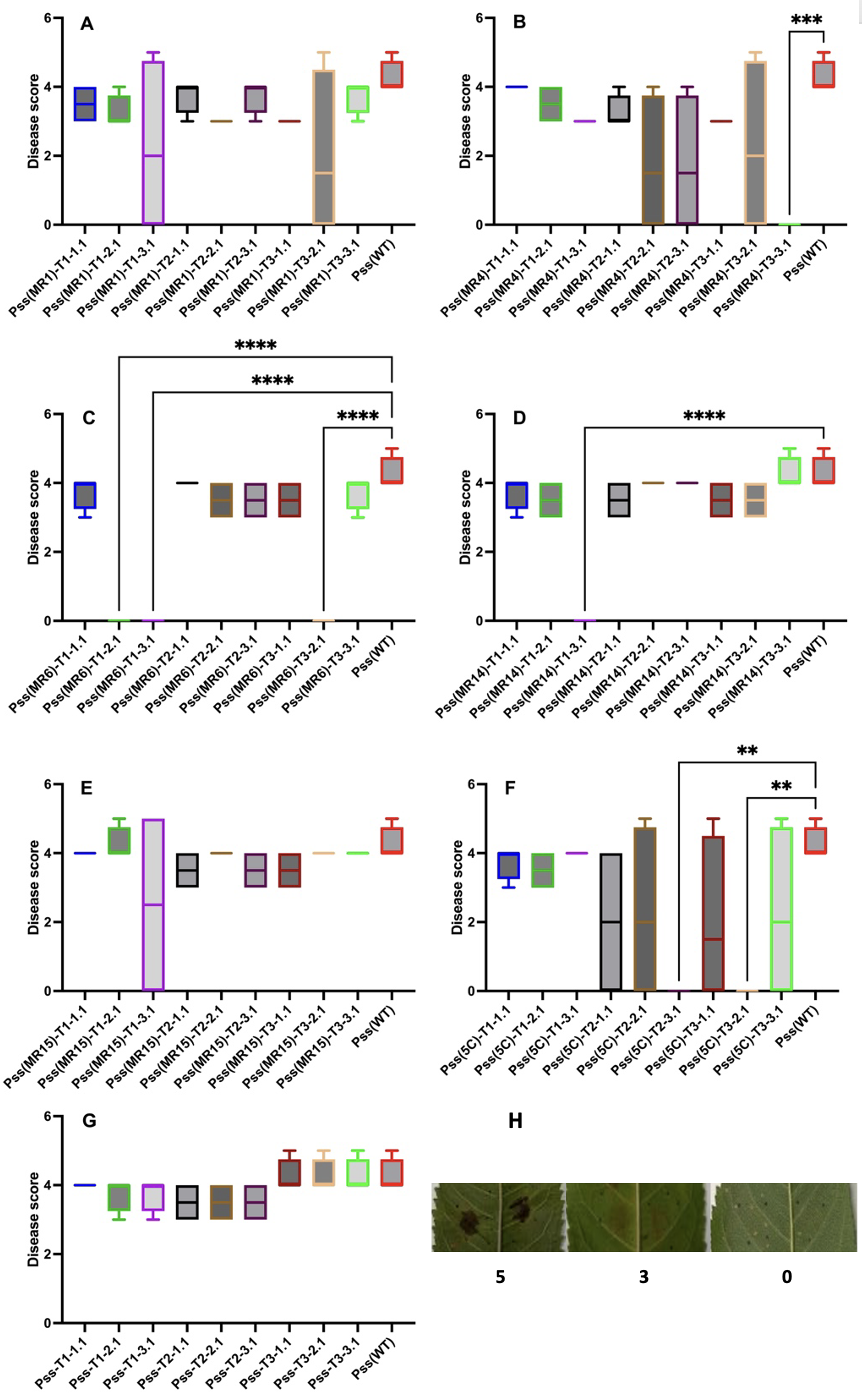


**Figure S5**. **Disease score of** ***Pseudomonas* *syringae* pv. *syringae* strain 9097 (*Pss*) phage-resistant isolates after infiltration in cherry leaves.** *Pss* isolates grown with phage MR1 (**A**), MR4 (**B**), MR6 (**C**), MR14 (**D**), MR15 (**E**), 5C (mixture of 5 phage) (**F**) and *Pss* with no phage (**G**), collected at T1, T2 and T3 from a 66 h *in vitro* experiment were infiltrated into cherry cultivar Sweetheart leaves (3 replicates) and the leaves incubated at room temperature. Disease symptoms were scored after 7d with 5 being 100% browning to 0 with no symptoms (**H**). *Pss* wildtype 9097 (WT) was included as a control. A mock inoculation with PBS caused no symptom development or recovered bacterial colonies (data not shown). The box plots created in GrahPad Prism 9. Asterisks show significant differences P>0.005 after ANOVA analysis.

**Figure S6**. **Population counts of** ***Pseudomonas* *syringae* pv. *syringae* strain 9097 (*Pss*) phage-resistant isolates in cherry leaves.** *Pss* isolates grown with phage MR1 (**A**), MR4 (**B**), MR6 (**C**), MR14 (**D**), MR15 (**E**), 5C (mixture of 5 phage) (**F**) and *Pss* with no phage (**G**), collected at T1, T2 and T3 from a 66 h *in vitro* experiment were infiltrated into cherry cultivar Sweetheart leaves (3 replicates) and the leaves incubated at room temperature. *Pss* wildtype 9097 (WT) was included as a control. A mock inoculation with PBS caused no symptom development or recovered bacterial colonies (data not shown). The violin plots created in GrahPad Prism 9 show median (dashed horizontal line), 25^th^ and 75^th^ percentiles (dotted horizontal lines, where visible) and lower and upper adjacent values. Asterisks show significant differences P>0.005 after ANOVA analysis.

**Figure S7**. **Disease score of** **phage-coevolved *Pseudomonas* *syringae* pv. *syringae* strain 9097 (*Pss*) mutants after infiltration in cherry leaves.** *Pss* isolates coevolved with phage MR1 (**A**), MR4 (**B**), MR6 (**C**), MR14 (**D**), MR15 (**E**), 5C (mixture of 5 phage) (**F**) and collected at 2^nd^ (2B), 6^th^ (6B) and 10^th^ (10B) transfer were infiltrated into cherry cultivar Sweetheart leaves (3 replicates) and the leaves incubated at room temperature. Disease symptoms were scored after 7d with 5 being 100% browning to 0 with no symptoms (See Figure S4). *Pss* wildtype 9097 (WT) was included as a control. A mock inoculation with PBS caused no symptom development or recovered bacterial colonies (data not shown). The box plots created in GrahPad Prism 9. ANOVA analysis found no significant differences between the wildtype *Pss* and other treatments were observed.

**Figure S8**. **Population count of** **phage-coevolved *Pseudomonas* *syringae* pv. *syringae* strain 9097 (*Pss*) mutants in cherry leaves.** *Pss* isolates coevolved with phage MR1 (**A**), MR4 (**B**), MR6 (**C**), MR14 (**D**), MR15 (**E**), 5C (mixture of 5 phage) (**F**) and collected at 2^nd^ (2B), 6^th^ (6B) and 10^th^ (10B) transfer were infiltrated into cherry cultivar Sweetheart leaves (3 replicates) and the leaves incubated at room temperature. *Pss* wildtype 9097 (WT) was included as a control. A mock inoculation with PBS caused no symptom development or recovered bacterial colonies (data not shown). The violin plots created in GrahPad Prism 9 show median (dashed horizontal line), 25^th^ and 75^th^ percentiles (dotted horizontal lines, where visible) and outliers lower and upper adjacent values. Asterisks show significant differences P>0.005 after ANOVA analysis.


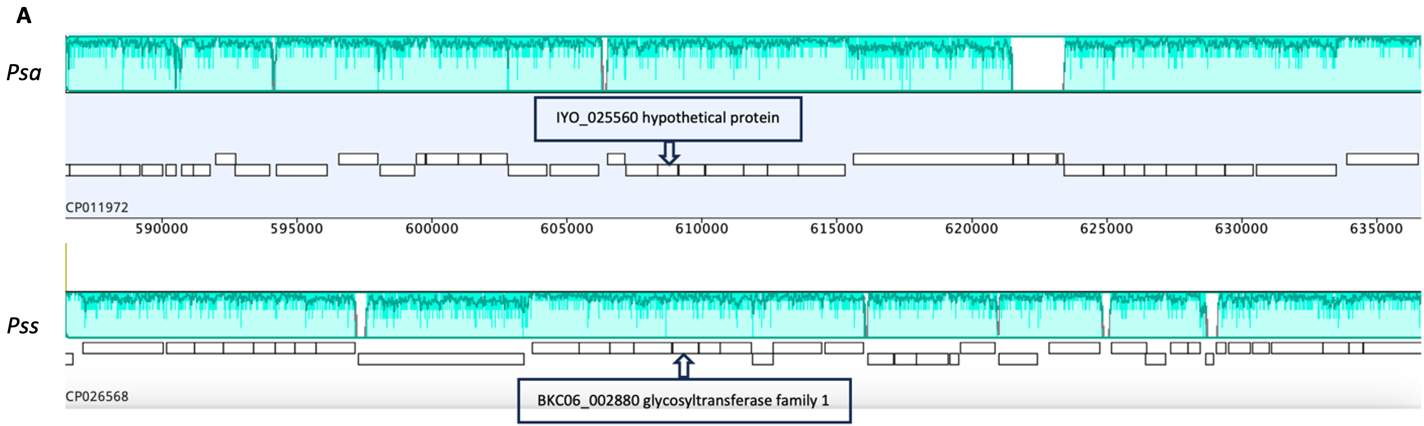


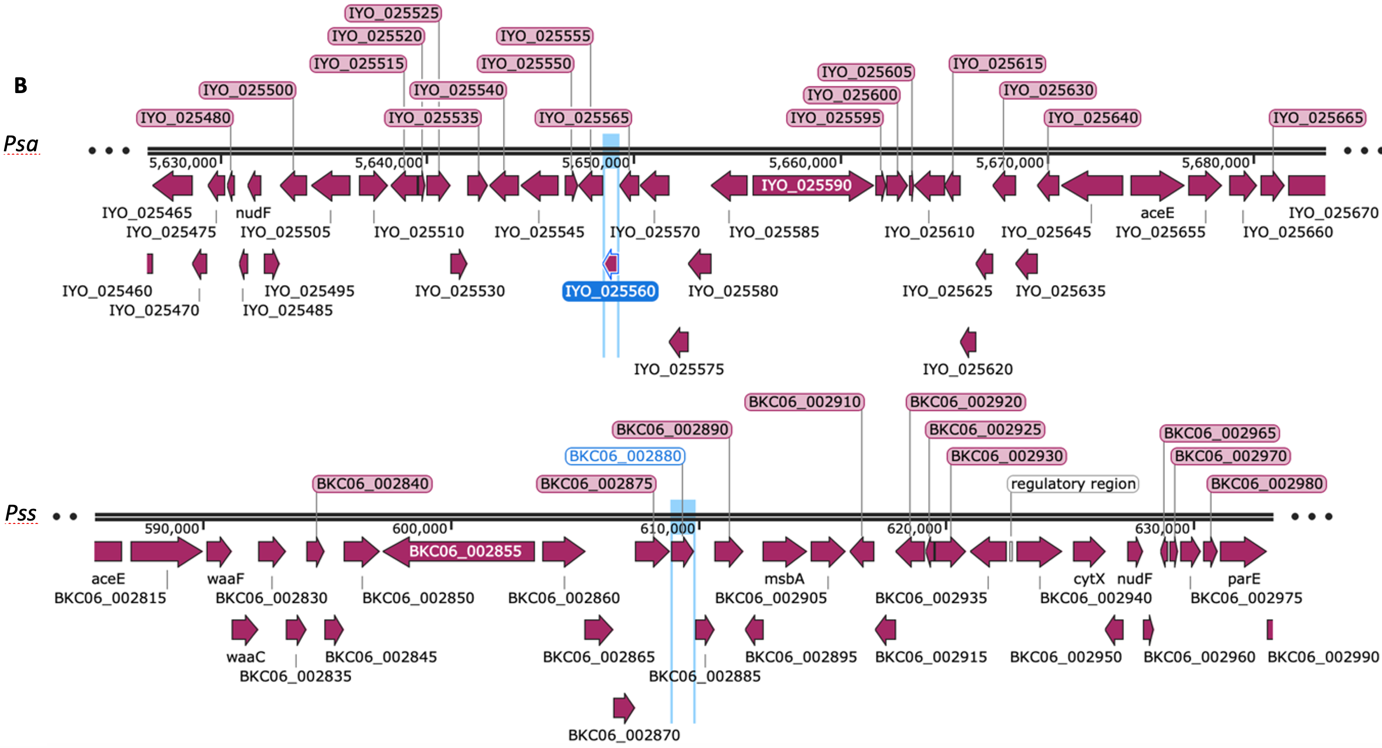


**Figure S9**. **Comparison of Lipopolysaccharide (LPS) gene cluster in *Pseudomonas syringae* pv. *actinidiae* ICMP 18884 (*Psa*, accession number CP011972) and *P. syringae* pv. syringae strain 9097 (*Pss*, accession number CP026568).** **A**. Alignment of the hypothetical protein, locus_tag IYO_025560 (known as glycosyltransferase family 2 protein) in *Psa* and glycosyltransferase family 1 protein gene, locus_tag BKC06_002880 in *Pss*. The alignment was done in geneious primer version 2023.2.1 using Mauve Genome progressive algorithm (default parameters). **B.** LPS gene cluster in both *Psa* and *Pss*, generated in SnapGene version 7.1.
